# Efficacy and safety of glucagon-like peptide-1 receptor agonists on prediabetes: a systematic review and meta-analysis of randomized controlled trials

**DOI:** 10.1186/s13098-024-01371-3

**Published:** 2024-06-14

**Authors:** Hazem Mohamed Salamah, Ahmed Marey, Mohamed Abugdida, Khaled Alsayed Abualkhair, Salem Elshenawy, Wael Atif Fadl Elhassan, Mostafa Mahmoud Naguib, Dmitrii Malnev, Jamrose Durrani, Ronelle Bailey, Anastasiia Tsyunchyk, Lena Ibrahim, Zhanna Zavgorodneva, Andleeb Sherazi

**Affiliations:** 1 Faculty of Medicine, Zagazig University, Zagazig, 44519 Egypt; 2 Faculty of Medicine, Alexandria University, Alexandria, Egypt; 3 Faculty of Medicine, Minia University, Minia, Egypt; 4 Faculty of Medicine, University of Khartoum, Khartoum, Sudan; 5 Faculty of Medicine, Al-Azhar University, Damietta, Egypt; 6 Department of Internal Medicine, Brookdale University Hospital and Medical Center, Brooklyn, NY USA

**Keywords:** Diabetes, Liraglutide, Prediabetes, Reversion, Regression, Normoglycemia, Impaired, Tolerance, Lifestyle

## Abstract

**Background:**

Prediabetes is a condition preceding the development of diabetes and is associated with an increased risk of a number of complications. The primary mode of management is thought to be lifestyle modification. Pharmacological therapy, such as glucagon-like peptide-1 receptor agonists (GLP-1RAs), were not well addressed in the literature and were only evaluated in trials as secondary and exploratory outcomes with a limited sample size. Here, GLP-1RAs are evaluated as a comprehensive therapy approach for patients with prediabetes.

**Methods:**

A comprehensive search of Web of Science, SCOPUS, PubMed, and Cochrane was performed on May 5, 2023, to retrieve randomized controlled trials (RCTs) comparing the effect of GLP-1RAs to placebo and/or lifestyle modification on prediabetes reversion to normoglycemia, prevention of overt diabetes, glycemic control, anthropometric parameters, and lipid profiles. Review Manager (RevMan) version 5.4 was used. The quality of RCTs was assessed using the revised version of the Cochrane Risk of Bias Tool. GRADE was performed to evaluate the certainty of evidence.

**Results:**

Twelve trials involving 2903 patients in the GLP-1RAs group and 1413 in the control group were included in the meta-analysis. Low quality of evidence revealed that GLP-1RAs significantly increased the incidence of prediabetes reversion to the normoglycemic state [RR = 1.76, 95% CI (1.45, 2.13), P < 0.00001] and moderate quality of evidence showed that GLP-1RAs significantly prevented new-onset diabetes [RR = 0.28, 95% CI (0.19, 0.43), P < 0.00001]. Significant reductions in HbA1c, fasting plasma glucose, body weight, waist circumference, triglycerides, and LDL were observed in the GLP-1RAs arm (P < 0.05). However, higher incidences of gastrointestinal disorders were reported in the GLP-1RAs group (P < 0.05).

**Conclusions:**

GLP-1RAs combined with lifestyle modification proved to be a more effective therapy for managing prediabetic patients than lifestyle modification alone, with a tolerable safety profile. Future guidelines should consider GLP-1RAs as an adjunct to lifestyle modification in the management of prediabetic patients to provide better management and improve treatment adherence.

**Supplementary Information:**

The online version contains supplementary material available at 10.1186/s13098-024-01371-3.

## Background

Prediabetes is a condition characterized by impaired glucose tolerance and/or fasting glucose levels that are higher than normal but not high enough to be diagnosed as diabetes [1]. Prediabetes is defined by the World Health Organization (WHO) as impaired glucose tolerance (IGT), impaired fasting glucose (IFG), or a combination of the 2 [2]. IGT is plasma glucose of 7.8–11.0 mmol/L (140–200 mg/dL) two hours after ingestion of 75 g of glucose. IFG is defined as fasting plasma glucose (FPG) levels ranging from 6.1–6.9 mmol/L (110–125 mg/dL) [2]. The American Diabetes Association (ADA) uses the same criteria but with a lower cut-off value for IFG (100–125 mg/dL) and adds HbA1c level of 5.7% to 6.4% to define prediabetes [3].

According to the impaired fasting glucose criteria, around 10.6% of adults globally (541 million individuals) are estimated to have prediabetes in 2021, which will increase to 11.4% (730 million people) by 2045 [4]. Prediabetes progresses to diabetes in 25% of cases within 3–5 years, and 70% of prediabetics develop overt diabetes in their lifetime [1]. In addition to an increased risk of diabetes progression and diabetes-related complications, prediabetes is associated with an increased risk of cardiovascular disease, chronic kidney disease, retinopathy, and other complications [1, 5–8].

Lifestyle interventions, such as diet and exercise, play a pivotal role in preventing or delaying the progression of prediabetes to diabetes [9, 10]. Their implementation and long-term adherence, however, pose significant challenges. Diabetes prevention program revealed that adherence to lifestyle interventions was lower than adherence to metformin. While only 38% of patients in the lifestyle group retained their weight loss, medication adherence over 4 years was around 70% [11]. This highlights the importance of investigating alternative strategies for managing prediabetes, such as pharmacological interventions.

Glucagon-like peptide-1 receptor agonists (GLP-1RAs) are a class of drugs that mimic the effects of GLP-1, a hormone that stimulates insulin secretion, inhibits glucagon secretion, delays gastric emptying, and reduces appetite [12]. GLP-1RAs stimulate GLP-1 receptors in the pancreas, increasing insulin release and alleviating hyperglycemia. Stimulation of GLP-1 receptors in the hypothalamus reduces appetite and increases satiety, which aids in weight loss [13]. A 5% to 7% weight loss can significantly reduce the risk of type 2 diabetes [14, 15]. GLP-1 receptor agonists have been shown to improve glycemic control, decrease body weight, and cardiometabolic parameters [16–18], improve atherosclerotic risk [19], lower cardiovascular risk [20], improve endothelial dysfunction [21], and improve dyslipidemia [22]. Furthermore, GLP-1 receptor agonists showed positive effects on factors that trigger prediabetes, such as oxidative stress [23, 24] and inflammation [24, 25]. Therefore, GLP-1RAs may be a viable intervention for the management of prediabetes.

The effect of GLP-1RAs on patients with impaired glucose tolerance has not been properly addressed in the literature. Several trials explored the effect of GLP-1RAs inhibitors on preventing the development of type 2 diabetes in prediabetic subjects as secondary or exploratory analyses [26–29]; however, some of the results were not statistically significant, which may be due to small sample sizes.

In this systematic review and meta-analysis, we aim to address this gap in knowledge by evaluating the efficacy and safety of GLP-1 receptor agonists in patients with prediabetes. We will also examine their effects on other diabetes-related parameters, such as HbA1c, fasting plasma glucose (FPG), hypoglycemia, body weight, and lipids. We will conduct subgroup analyses to examine the effect of different GLP-1RA treatment durations, types, and doses. The findings of this review have the potential to inform clinical practice, guidelines, and future research directions in the management of prediabetes.

## Methods

The authors followed the PRISMA guidelines for reporting systematic reviews and meta-analyses of randomized controlled trials (RCTs) [30]. This systematic review and meta-analysis was registered in the International Prospective Register of Systematic Reviews (PROSPERO) with registration ID CRD42023456814.

### Eligibility criteria

Studies were included if they met the PICOS criteria: patients, intervention, control, outcomes, and study design. The patients of interest were prediabetic or had impaired glucose tolerance or impaired fasting plasma glucose with or without obesity or overweight. The intervention was GLP-1RAs or dual glucose-dependent insulinotropic polypeptide (GIP)/GLP-1 receptor agonists such as tirzepatide, alone or combined with lifestyle modification. The control group included prediabetic patients who received a placebo and/or lifestyle modification. The studies must measure and report the results of the outcomes of interest separately for the prediabetic patients to be included. The authors included only randomized controlled trials comparing. If multiple publications exist for a single eligible study, they will be included if they provide new data, such as results for more treatment durations; otherwise, only the publication with the most data about prediabetic patients and their baseline data will be included.

Non-randomized trials, animal studies, conference abstracts, non-English papers, and single-arm studies were all excluded. Studies in which both diabetic and prediabetic patients were included, but the results of the prediabetic patients were not reported separately were excluded. Studies in which patients had polycystic ovary syndrome or received GLP-1RAs in combination with other drugs that raise blood glucose levels were excluded.

### Information sources

PubMed, Web of Science, Scopus, and Cochrane were searched for the relevant articles on February 20, 2023, and we updated the search again on May 5, 2023. The references of the eligible papers were also searched for other relevant studies.

### Search strategy

PubMed, Web of Science, Scopus, and Cochrane were searched using a combination of the following terms: “Glucagon Like Peptide”, “Prediabetic”, “State” “Trial”, “Exenatide”, “Semaglutide”, “Efpeglenatide”, “lixisenatide”, “Liraglutide”, and “Tirzepatide”. No filters were applied. The full search strategy is shown in Table S1.

### Selection process

Endnote was used to gather articles from various databases. Afterward, the articles were exported to an Excel spreadsheet and screened in 2 stages for studies that met our inclusion criteria. The first stage entailed screening the title and abstract of the retrieved records, with those who passed progressing to the second stage, which entailed screening the full text. Two independent reviewers carried out the screening, and disagreements were settled through discussion or by a third author.

### Data collection process

The lead author prepared formatted Excel sheets for extracting patients’ baseline data, study characteristics, risk of bias (ROB) assessment, and outcomes of interest. The two authors extracted data from each study independently and then discussed it. A third senior author settled any disagreements. Methods recommended in the Cochrane Handbook [31] were used to deal with any incomplete or incompatible data.

### Data items (outcomes)

The primary outcomes were the incidence of prediabetes reversion to normoglycemia and the incidence of developing overt diabetes. The secondary outcomes were fasting plasma glucose (FPG), HbA1c, weight loss, waist circumference, lipid profile outcomes, which are triglycerides (TG), low-density lipoprotein (LDL), and high-density lipoprotein (HDL), and safety outcomes, which are any adverse event (AEs), any serious AEs, any gastrointestinal disorders, nausea, vomiting, diarrhea, hypoglycemia, and headache.

### Data items (other variables)

Two authors independently extracted study characteristics and baseline data. The characteristics of the studies included study ID, country and center, criteria of prediabetes diagnosis, inclusion and exclusion criteria, name and dose of the intervention and control, ample size, and follow-up duration. Patients baseline data included age, gender, weight, BMI, fasting plasma glucose (FPG), HbA1c, TG, and LDL.

#### Study risk of bias assessment

The Cochrane risk-of-bias tool version 2 [32] was used to assess the quality of the included studies in the following domains: (A) bias arising from the randomization process, B) bias resulting from deviations from intended interventions, (C) bias resulting from missing outcome data, (D) bias in outcome measurement, and (E) bias in the selection of the reported results. The domains were classified as low, moderate, or high risk. Two authors conducted the evaluation independently, with discussions with a third author in the event of disagreements. The Grading of Recommendations Assessment, Development, and Evaluation (GRADE) criteria were used to evaluate the quality of evidence.

### Effect measures and synthesis methods

Review Manager (RevMan) version 5.4 [33] was used to conduct all the analyses. Continuous data were extracted as means and standard deviation (SD) or mean difference against placebo and standard error, while dichotomous data were extracted as event and total. The dichotomous outcomes, including the incidence of prediabetes reversion to normoglycemia, the incidence of new-onset diabetes, and safety outcomes, were pooled using the Mantel-Haensze equation and reported as risk ratio (RR) and 95% confidence interval (CI). The continuous outcomes, including FPG, HbA1c, body weight reduction, waist circumference, TG, HDL, and LDL, were pooled using the generic inverse variance statistic method and reported as mean differences with a 95% CI.

We conducted subgroup analysis based on the treatment duration and type and dose of the GLP-1RAs whenever the number of studies included in the analysis was sufficient.

Cochrane's Q test and the I2 statistic were used to assess heterogeneity. If the P value was less than 0.1, the heterogeneity was considered significant, and a random-effects model was used; otherwise, a fixed-effects model was used. A sensitivity analysis using the leave-one-out method was used to identify the source of heterogeneity and investigate the robustness of our results.

TSA was performed with a 5% risk of type I error and a 10% risk of type II error (90% power). The TSA was conducted in chronological order by year of publication. TSA was performed using the TSA Viewer, version 0.9 beta (Copenhagen Trial Unit, Copenhagen, Denmark).

A funnel plot, as well as Egger's and Begg's tests, were used to investigate publication bias, and P < 0.05 was judged significant [34, 35]. Trim-and-fill statistical analysis was used to account for potential publication bias [36].

## Results

### Literature search results

**Fig. 1:**
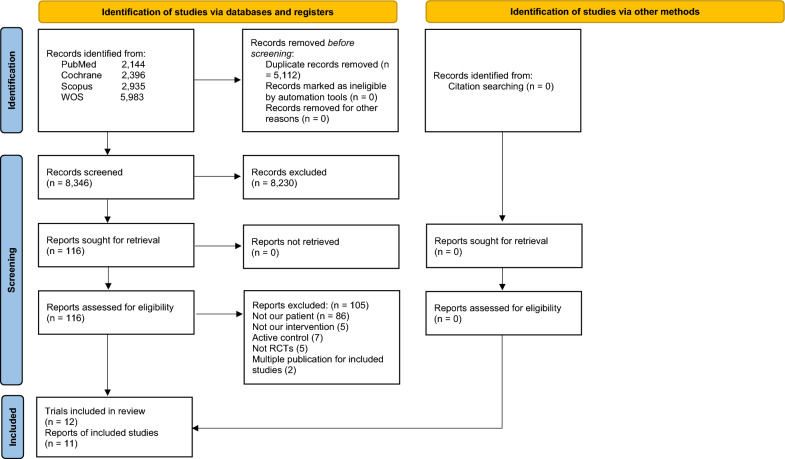
PRISMA flow diagram shows the detailed process of search strategy results and study selection

The search produced 13,458 results. After duplicates were removed, the total number was 8,346. Only 116 papers were eligible for full-text screening after title and abstract screening. Finally, 12 trials with a total of 11 publications [26–29, 37–43] were determined to be eligible for the final analysis. The paper of Perreault et al. 2022 [28] reported the results of the outcomes of interest for prediabetic patients from three trials. Astrup et al. [37] and [42] were two publications for one trial. Figure 1 demonstrates a PRISMA flow diagram.

### Study characteristics

The meta-analysis included a total of 2903 prediabetic patients in the GLP-1RAs group and 1,413 in the control group. Most of the included studies were international multicenter studies [26, 28, 37, 39, 42], while six studies were single-center: three were conducted in the United States [40, 41, 43], one in China [38], and one in Sweden [27]. Of the GLP-1RAs used, Liraglutide was administered in six trials: Astrup et al. [37, 42] examined four doses of 1.2 mg, 1.8 mg, 2.4 mg, or 3.0 mg once/day, while three trials investigated dose 1.8 mg/day [40, 41, 43], one trial investigated dose 1.2 mg/day [38], and the remaining trial gave 3 mg/day [26]. Semaglutide at a dose of 2.4mg QW was given in the three trials published in the paper of Perreault et al. 2022 [28]. Efpeglenatide at doses of 4 mg QW, 6 mg QW, 6 mg Q2W, or 8 mg Q2W was administered in Pratley et al. 2021 [39]. Exenatide at a dose of 10 µg twice a day was administered in one study [29] and at a dose of 2 mg QW combined with Dapagliflozin in another study [27]. All studies gave GLP-1RAs in combination with lifestyle modification, except Mashayekhi et al. 2022 [40], where they compared the GLP-1RAs alone to the hypocaloric diet. The detailed characteristics of the included studies and the follow-up durations are shown in Table 1. All studies included obese patients ≥ 18 years old, except Zhou et al. 2017, which included obese prediabetic children. The inclusion and exclusion criteria applied by each study are shown in Table S2. The mean BMI of the included patients in all studies was > 30 kg/m^2^. Patients’ baseline characteristics are shown in Table 2.

**Table 1:** Study characteristics

Study ID	Country/Center	Criteria for prediabetes diagnosis	Definition of new-onset diabetes	Prediabetic sample size/Total sample size	Follow-up duration	Intervention group	Control group	Comedications status
Ariel et al. 2014	USA (Single-center)	FPG ≥ 100 and < 126 mg/dL and/or a plasma glucose concentration ≥ 140 and < 190 mg/dL 2-h after a 75-g oral glucose challenge	NA	68/68	14 weeks	Liraglutide, 1.8 mg/day, SC, starting at 0.6 mg daily, increased by 0.6 mg weekly to 1.8 mg/day + calorie-restricted diet	Placebo + calorie-restricted diet	Patients were excluded if they were taking any medications that affect lipoprotein or carbohydrate metabolism or promote weight loss
Rosenstock et al. 2010	USA	IGT (fasting glucose < 7 mmol/L and 2-h postprandial glucose ≥ 7.8 and < 11.1 mmol/L), IFG (fasting glucose 6.1– 6.9 mmol/l and 2-h postprandial glucose < 7.8 mmol/l)	NA	33/152	24 weeks	Exenatide, 10 µg, twice/day + a structured program of diet and physical activity	Placebo + a structured program of diet and physical activity	Patients with previous use of glucose-lowering medications for more than 3 months were excluded
Mashayekhi et al. 2022	USA (Single-center)	FPG between 100–125 mg/dL or IGT after a 75-g glucose challenge of 140–199 mg/dL or HbA1c = 5.7 to 6.4%	NA	88/88	14 weeks	Liraglutide, 1.8 mg/day, SC, starting at 0.6 mg daily, increased by 0.6 mg weekly to 1.8 mg/day	Sitagliptin 100 mg/day or a hypocaloric diet	NA
Pratley et al. 2021	Five countries (Multicenter)	HbA1c = 5.7% to 6.4% or FPG = 100 to 125 mg/dL	NA	140/140	20 weeks	Efpeglenatide, 4 mg QW, 6 mg QW, 6 mg Q2W, or 8 mg Q2W, SC + reducing calorie intake by approximately 500 kcal each day and increasing physical activity	Placebo + reducing calorie intake by approximately 500 kcal each day and increasing physical activity	NA
Kim et al. 2013	USA (Single-center)	FPG = 5.6 to 6.9 mmol/L or elevated 2-h glucose (7.8–11.0 mmol/L) concentration after a 75 g OGTT	NA	68/68	14 weeks	Liraglutide, 1.8 mg/day, SC, starting at 0.6 mg daily, increased by 0.6 mg weekly to 1.8 mg/day + decreasing total caloric intake by 500 kcal/day, and keeping baseline physical activity	Placebo + decreasing total caloric intake by 500 kcal/day and keeping baseline physical activity	Patients using medications that affect carbohydrate metabolism or promote weight loss were excluded
Lundkvist et al. 2017	Sweden (Single-center)	Prediabetes was defined as any IFG or IGT. IFG (FPG ≥ 5.6 mmol/L measured just before the OGTT), IGT ≥ 7.8 mmol/L measured 120 min after the start of the OGTT)	FPG > 7.0 mmol/L, 2-h OGTT > 11.1 mmol/L, and/or HbA1c > 48 mmol/mol	33/49	24 weeks	Dapagliflozin 10 mg, once/day, oral + Exenatide 2 mg QW, SC + balanced diet and moderate exercise. However, diet and exercise modifications were not mandated or documented	Placebo + balanced diet and moderate exercise. However, diet and exercise modification were not mandated or documented	Remained on the same medication without alternations
Zhou et al. 2017	China (Single-center)	IFG: FPG (5.6–6.9 mmol/L), while 2 h OGTT < 7.8 mmol/L, IGT: OGTT 2 h blood glucose (7.8–11.0) while FPG < 5.6	FPG ≥ 7.0 or OGTT 2 h blood glucose > 11.1	42/42	12 weeks	Liraglutide, 1.2 mg/day, SC, starting at 0.6 mg daily, increased to 1.2 mg/day after one week + a unified diet and exercise prescription with regular telephone conversations and outpatient follow-up	A unified diet and exercise prescription with regular telephone conversations and outpatient follow-up	NA
Astrup et al. 2009	19 clinical research sites in eight European countries	Either IFG (5·6–6·9 mmol/L) or IGT (7·8–11·0 mmol/L) during OGTT	Fasting plasma glucose ≥ 7·0 mmol/L or ≥ 11·1 mmol/L during OGTT	187/564	20 weeks	Liraglutide, 1.2 mg, 1.8 mg, 2.4 mg, or 3.0 mg, once/day, SC + decreasing total caloric intake by 500 kcal/day, and increasing physical activity	Placebo or orlistat + decreasing total caloric intake by 500 kcal/day, and increasing physical activity	Patients were excluded weight-lowering pharmacotherapy or participation in a clinical weight control study
Astrup et al. 2012	19 clinical research sites in eight European countries	Either IFG (5·6–6·9 mmol/L) or IGT (7·8–11·0 mmol/L) during OGTT	Fasting plasma glucose ≥ 7·0 mmol/L or ≥ 11·1 mmol/L during OGTT	187/564	52 weeks	Liraglutide, 1.2 mg, 1.8 mg, 2.4 mg, or 3.0 mg, once/day, SC + decreasing total caloric intake by 500 kcal/day, and increasing physical activity	Placebo or orlistat + decreasing total caloric intake by 500 kcal/day, and increasing physical activity	Patients were excluded if they used weight-lowering pharmacotherapy or participation in a weight-control stud
Le Roux et al. 2017	191 clinical research sites in 27 countries	One of the three American Diabetes Association (ADA) 2010 criteria: HbA1c = 5·7 − 6·4%, or FPG = 5·6 mmol/L to 6·9 mmol/L, or 2-h OGTT = 7·8 mmol/L to 11·0 mmol/L	Two consecutive measurements of HbA1c ≥ 6.5%, or FPG ≥ 126 mg/dl (7.0 mmol/liter), or 2-h OGTT ≥ 200 mg/dl (11.1 mmol/liter)	2254/2254	160 weeks	Liraglutide, 3.0 mg, once/day, SC, starting at 0.6 mg with weekly 0.6-mg increments to 3.0 mg + reduced-calorie diet, and increased physical activity	Placebo + reduced-calorie diet, and increased physical activity	Patients were excluded medications causing significant weight gain or loss
Perreault et al. 2022a	16 countries (multicenter)	American Diabetes Association criteria. Prediabetes was defined as FPG 5.6–6.9 mmol/L or HbA1c 5.7–6.4% (39–47 mmol/mol)	HbA1c = 48 mmol/L (6.5%) or greater	856/1961	68 weeks	Semaglutide, 2.4 mg, QW, SC, starting with 0.25 mg weekly for the first 4 weeks, increased every 4 weeks to reach 2.4 mg weekly + decreasing total caloric intake by 500 kcal/day, and increasing physical activity by 150 min/week of physical activity. Both diet and activity were recorded daily	Placebo + decreasing total caloric intake by 500 kcal/day, and increasing physical activity. Both diet and activity were recorded daily	The individual has not had any prior surgical obesity treatment or use of antiobesity medication within 90 days prior to enrollment
Perreault et al. 2022b	USA (multicenter)	American Diabetes Association criteria. Prediabetes was defined as FPG 5.6–6.9 mmol/L or HbA1c 5.7–6.4% (39–47 mmol/mol)	HbA1c = 48 mmol/L (6.5%) or greater	304/611	68 weeks	Semaglutide, 2.4 mg, QW, SC, starting with 0.25 mg weekly for the first 4 weeks, increased every 4 weeks to reach 2.4 mg weekly + intensive behavioral intervention (low-calorie diet (1000–1200 kcal/d) in the initial 8 weeks + hypocaloric diet (1200–1800 kcal/d) for the rest of the 68 weeks) + 100 min of physical activity per week, which increased by 25 min every 4 weeks to reach 200 min/week	Placebo + intensive behavioral intervention (low-calorie diet (1000–1200 kcal/d) in the initial 8 weeks + hypocaloric diet (1200–1800 kcal/d) for the rest of 68 weeks) + 100 min of physical activity per week, which increased by 25 min every 4 weeks, to reach 200 min/week	NA
Perreault et al. 2022c	10 countries (multicenter)	American Diabetes Association criteria. Prediabetes was defined as FPG 5.6–6.9 mmol/L or HbA1c 5.7–6.4% (39–47 mmol/mol)	HbA1c = 48 mmol/L (6.5%) or greater	376/803	48 weeks	Semaglutide, 2.4 mg, QW, SC, starting with 0.25 mg weekly for the first 4 weeks, increased every 4 weeks to reach 2.4 mg weekly + decreasing total caloric intake by 500 kcal/day, and increasing physical activity by 150 min/week of physical activity. Both diet and activity were recorded daily	Placebo + decreasing total caloric intake by 500 kcal/day, and increasing physical activity. Both diet and activity were recorded daily	NA

IGT: impaired glucose tolerance; IFG: impaired fasting plasma glucose; FPG: fasting plasma glucose; OGTT: oral glucose tolerance test; SC: subcutaneous; QW: once weekly; Q2W: once every 2 weeks

**Table 2:** Patients baseline characteristics

Study ID	Groups	Sample size	Age (years)	Female	Weight (kg)	BMI (kg/m^2^)	HbA1c (%)	FPG (mg/dl)	TG (mg/dl)	LDL (mg/dl)
			Mean ± SD	N (%)	Mean ± SD	Mean ± SD	Mean ± SD	Mean ± SD	Mean ± SD	Mean ± SD
Ariel et al. 2014	Liraglutide 1.8 mg daily	23	58 ± 7	15 (67)	88.9 ± 11.2	31.9 ± 2.8	NA	105 ± 8	144.4 ± 73.7	113.3 ± 25.9
	Placebo	27	58 ± 8	17 (63)	88.7 ± 11.2	31.9 ± 3.5	NA	107 ± 8	129.8 ± 62.6	117.4 ± 31.5
Rosenstock et al. 2010 **#**	Exenatide 10ug BID	73	46 ± 12	124 (82)	109.5 ± 2.7	39.6 ± 7.0	NA	NA	NA	NA
	Placebo	79			107.6 ± 2.6		NA	NA	NA	NA
Mashayekhi et al. 2022	Liraglutide 1.8 mg daily	44	49.8 ± 10.1	31 (70.5)	108.8 ± 20.9	38.8 ± 6.1	5.7 ± 0.3	96.2 ± 10.1	122.2 ± 51.4	119.1 ± 31.9
	Hypocaloric diet	22	49.2 ± 12.5	14 (63.6)	111.3 ± 21.5	38.4 ± 5.9	5.7 ± 0.3	96.7 ± 11.8	115.0 ± 67.1	111.5 ± 35.7
Pratley et al. 2021	Efpeglenatide 4 mg QW	28	45.6 ± 11.0	18 (64.3)	104.9 ± 22.0	35.9 ± 4.7	5.7 ± 0.3	100.3 ± 9.9	NA	NA
	Efpeglenatide 6 mg QW	26	46.2 ± 12.2	20 (76.9)	104.5 ± 23.3	36.8 ± 5.0	5.7 ± 0.3	101.7 ± 9.6	NA	NA
	Efpeglenatide 6 mg Q2W	32	48.5 ± 10.9	23 (71.9)	100.9 ± 19.5	35.9 ± 5.6	5.7 ± 0.3	103.4 ± 10.0	NA	NA
	Efpeglenatide 8 mg Q2W	24	47.5 ± 9.3	22 (91.7)	94.5 ± 11.3	34.8 ± 3.3	5.7 ± 0.3	103.3 ± 8.5	NA	NA
	Placebo	30	45.0 ± 11.3	23 (76.7)	96.9 ± 10.9	35.1 ± 3.1	5.7 ± 0.4	102.3 ± 12.0	NA	NA
Kim et al. 2013	Liraglutide 1.8 mg daily	24	58 ± 7	16 (67)	88.4 ± 11.2	31.9 ± 2.7	NA	106.2 ± 7.2	141.7 ± 70.9	112.14 ± 27.07
	Placebo	27	58 ± 8	17 (63)	88.7 ± 11.2	31.9 ± 3.5	NA	109.8 ± 7.2	132.9 ± 62	116 ± 30.94
Lundkvist et al. 2017 #	Dapagliflozin 10 mg daily + Exenatide 2 mg QW	25	53.3 ± 13.5	15 (60)	106.43 ± 15.55	35.8 ± 2.9	5.6 ± 0.35	106.2 ± 11.34	124 ± 50.49	136.12 ± 35.19
	Placebo	24	50 ± 11.8	15 (62.5)	102.72 ± 17.26	35 ± 3.7	5.6 ± 0.3	104.94 ± 7.74	140.83 ± 52.3	133.02 ± 34.42
Perreault et al. 2022a	Semaglutide 2.4 mg QW	593	48.5 ± 12.5	407 (68.6)	106.9 ± 22.4	38.4 ± 6.6	5.9 ± 0.2	98.7 ± 11.0	NA	NA
	Placebo	263	49.4 ± 12.1	196 (74.5)	106.9 ± 21.1	38.9 ± 6.5	5.9 ± 0.2	97.6 ± 11.8	NA	NA
Perreault et al. 2022b	Semaglutide 2.4 mg QW	196	48.8 ± 11.9	153 (78.1)	108.7 ± 22.9	39.0 ± 6.8	6.0 ± 0.2	96.8 ± 9.4	NA	NA
	Placebo	108	49.7 ± 12.3	88 (81.5)	106.9 ± 23.3	38.5 ± 6.9	6.0 ± 0.2	96.7 ± 9.2	NA	NA
Perreault et al. 2022c	Semaglutide 2.4 mg QW	262	49.9 ± 11.6	208 (79.4)	109.7 ± 25.1	39.3 ± 7.8	5.9 ± 0.2	100.9 ± 11.5	NA	NA
	Placebo	114	50.0 ± 10.6	81 (71.1)	109.6 ± 24.6	39.1 ± 7.6	5.9 ± 0.2	98.5 ± 9.3	NA	NA
Zhou et al. 2017	Liraglutide 1.2 mg daily	21	11.19 ± 2.27	7 (33)	NA	30.98 ± 4.97	5.87 ± 1.35	NA	100.09 ± 63.77	94.74 ± 38.67
	Lifestyle intervention	21	11.12 ± 2.10	6 (28.57)	NA	30.89 ± 5.02	5.85 ± 1.33	NA	100.97 ± 54.92	95.51 ± 37.12
Astrup et al. 2009 #	Liraglutide 1.2 mg daily	95	47·2 ± 9·7	73 (77)	96·2 ± 13·5	34·8 ± 2·6	5.6 ± 0.3	95.4 ± 10.8	127.55 ± 74.4	130.7 ± 29.776
	Liraglutide 1.8 mg daily	90	45·5 ± 10·9	68 (76)	98·0 ± 12·5	35·0 ± 2·6	5.6 ± 0.4	95.4 ± 10.8	126.66 ± 87.69	136.5 ± 34.03
	Liraglutide 2.4 mg daily	93	45·0 ± 11·1	70 (76)	98·4 ± 13·0	35·0 ± 2·8	5.5 ± 0.3	95.4 ± 10.8	121.35 ± 56.69	135.73 ± 33.26
	Liraglutide 3.0 mg daily	93	45·9 ± 10·7	70 (75)	97·6 ± 13·7	34·8 ± 2·8	5.6 ± 0.4	97.2 ± 10.8	125.78 ± 69.09	131.48 ± 30.16
	Placebo	98	45·9 ± 10·3	73 (75)	97·3 ± 12·3	34·9 ± 2·8	5.6 ± 0.4	97.2 ± 14.4	138.18 ± 69.09	136.5 ± 34.42
Le Roux et al. 2017	Liraglutide 3.0 mg daily	1505	47·5 ± 11·7	1141 (76)	107.5 ± 21.6	38.8 ± 6.4	5.8 ± 0.3	99 ± 10.8	132.86 ± 54.1	112.14 ± 27.9
	Placebo	747	47·3 ± 11·8	573 (77)	107.9 ± 21.8	39 ± 6.3	5.7 ± 0.3	99 ± 9	132.86 ± 66.6	116 ± 28

*QW* once per week; *Q2W* once every 2 weeks; *BID* twice per day

^#^data are for patients with and without prediabetes, as no data were reported for prediabetes separately at baseline

### Quality assessment

**Fig. 2:**
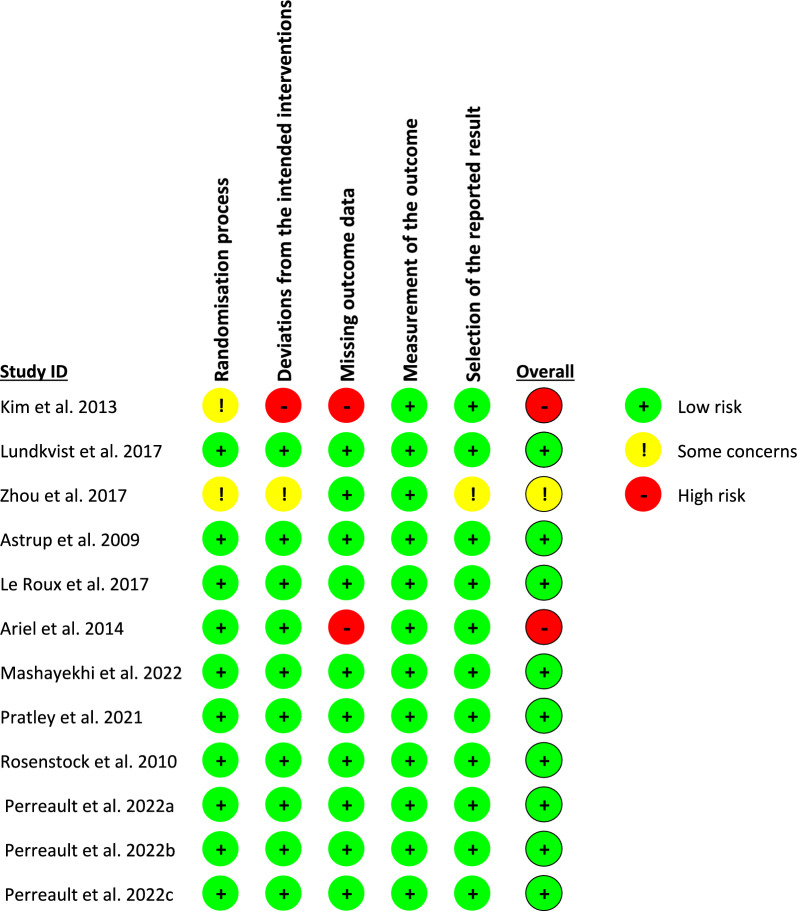
Summery of the risk of bias of the included studies

The quality of the included studies was evaluated using the revised Cochrane risk of bias tool, as shown in Fig. 2. Nine trials had a low risk of bias in all domains. Two trials showed a high risk of bias resulting from missing outcome data, and one of them showed a high risk of bias resulting from deviation from the intended intervention as well. Zhou et al. 2017 [38] showed some concern regarding the randomization process, deviation from the intended intervention, and selection of the reported results due to a lack of information. The quality of evidence was determined via GRADE instructions (Table S3).

## Primary outcomes

### The incidence of prediabetes reversion to normoglycemia

**Fig. 3:**
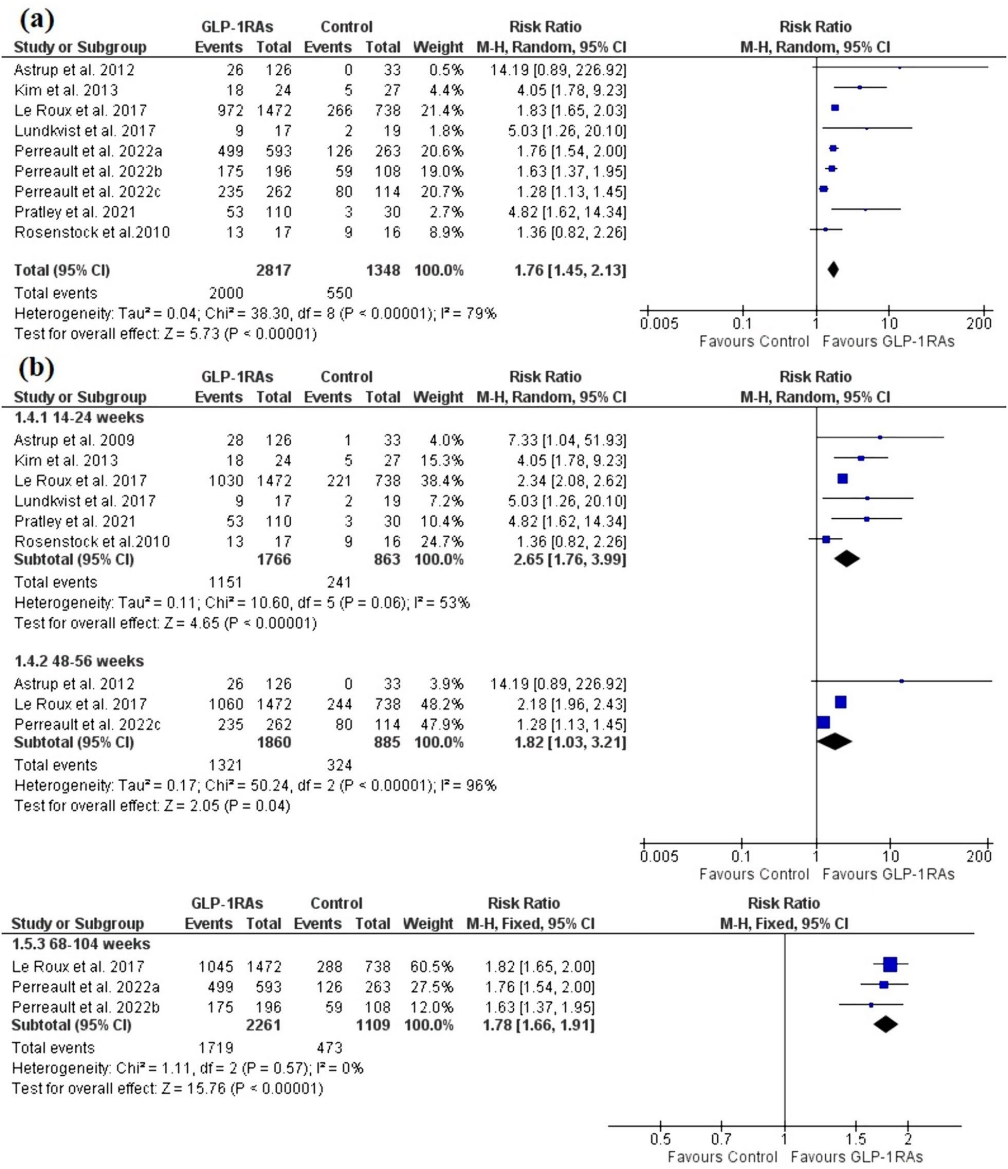
A forest plot shows the effect of GLP-1RAs on the incidence of prediabetes reversion to normoglycemic state. **a** Overall effect. **b** subgroup analysis based on the treatment duration

The analysis included nine trials with seven publications [26–29, 39, 42, 43] with a total of 2817 patients in the GLP-1RAs arm and 1348 patients in the control arm. Evidence with a low degree of certainty demonstrated that GLP-1RAs significantly revert prediabetic patients to a normoglycemia state compared to the control group [RR = 1.76, 95% CI (1.45, 2.13), P < 0.00001] (Fig. 3a). High heterogeneity was observed [I^2^ = 79%, P < 0.00001], which was partially resolved after the exclusion of Perreault et al. 2022c [I^2^ = 51%, P = 0.05] without significant change in the pooled analysis [RR = 1.83, 95% CI (1.57, 2.12), P < 0.00001].

Subgroup analysis revealed that the effect of GLP-1RAs on prediabetes reversion was accomplished as early as 14–24 weeks [RR = 2.65, 95% CI (1.76, 3.99), P < 0.00001] and lasted up to 48–56 weeks [RR = 1.82, 95% CI (1.03, 3.21), P = 0.04] and 68–104 weeks [RR = 1.78, 95% CI (1.66, 1.91), P < 0.00001] (Fig. 3b). The pooled analysis was homogenous for 68–104 weeks [I^2^ = 0%, P = 0.57], but heterogeneous for 14–24 weeks [I^2^ = 53%, P = 0.06] and 48–56 weeks [I^2^ = 96%, P < 0.00001], which was resolved after the exclusion of Rosenstock et al.2010 [I^2^ = 31%, P = 0.22], and Perreault et al. 2022c [I^2^ = 44%, P = 0.18], respectively, without significant change in the pooled analysis [RR = 2.44, 95% CI (2.18, 2.74), P < 0.00001], [RR = 2.21, 95% CI (1.98, 2.46), P < 0.00001].

Subgroup analysis revealed that semaglutide 2.4mg once weekly (QW) significantly outperformed the control group [RR = 1.54, 95% CI (1.24, 1.91), P < 0.00001]. The pooled analysis showed heterogeneity [I^2^ = 85%, P = 0.001]. Heterogeneity was eliminated by excluding Perreault et al. 2022c without significant change in the pooled analysis [RR = 1.71, 95% CI (1.54, 1.90), P < 0.00001]. Furthermore, Liraglutide 1.8mg once daily (QD) and 3mg were significantly effective [RR = 5.68, 95% CI (2.52, 12.82), P < 0.00001], [RR = 1.85, 95% CI (1.67, 2.05), P < 0.00001], and the pooled analyses were homogenous [I^2^ = 33%, P = 0.22], [I^2^ = 52%, P = 0.15] (Figure S1).

### The incidence of new-onset diabetes

**Fig. 4:**
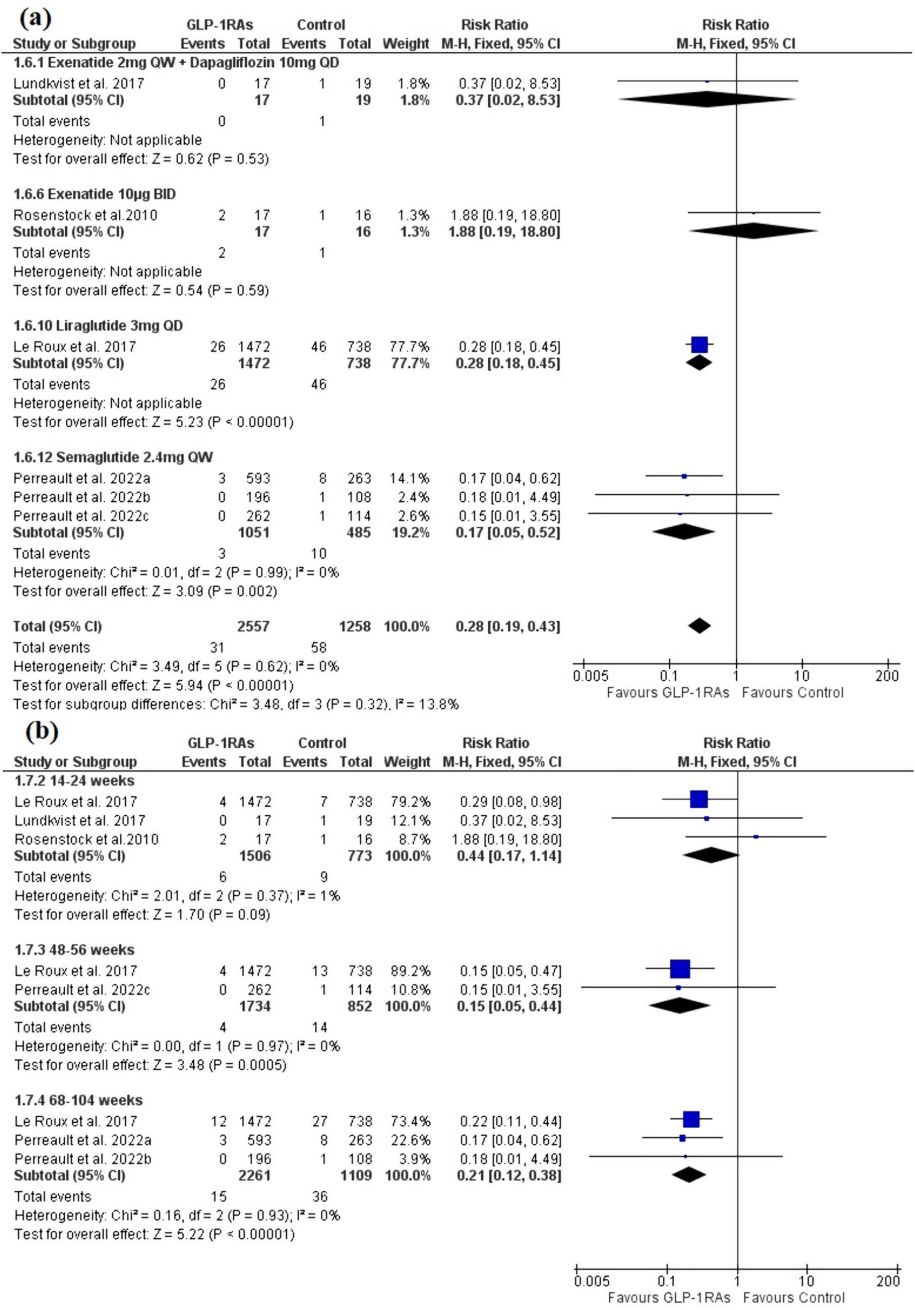
A forest plot shows the effect of GLP-1RAs on the incidence of new-onset diabetes. **a** Overall effect and subgroup analysis based on the type and dose of GLP-1RAs. **b** subgroup analysis based on the treatment duration

The analysis included six trials with four publications [26–29] with a total of 2557 patients in the GLP-1RAs arm and 1258 patients in the control arm. Evidence with a moderate degree of certainty demonstrated that prediabetic patients who progressed to a diabetes state were significantly fewer in the GLP-1RAs group compared to the control [RR = 0.28, 95% CI (0.19, 0.43), P < 0.00001]. Semaglutide 2.4mg QW significantly outperformed the control group in the subgroup analysis [RR = 0.17, 95% CI (0.05, 0.52), P = 0.002]. Both pooled analyses were homogenous [I^2^ = 0%, P = 0.62], and [I^2^ = 0%, P = 0.99] **(**Fig. 4a).

The protective effect of GLP-1RAs was noticed after 48–56 weeks and 68–104 weeks of the treatment [RR = 0.15, 95% CI (0.05, 0.44), P = 0.0005], [RR = 0.21, 95% CI (0.12, 0.38), P < 0.00001], but not as early as 14–24 weeks [RR = 0.44, 95% CI (0.17, 1.14), P = 0.09]. All the results were homogenous (P > 0.1) (Fig. 4b).

### Secondary outcomes

#### FPG (mg/dl)

**Fig. 5:**
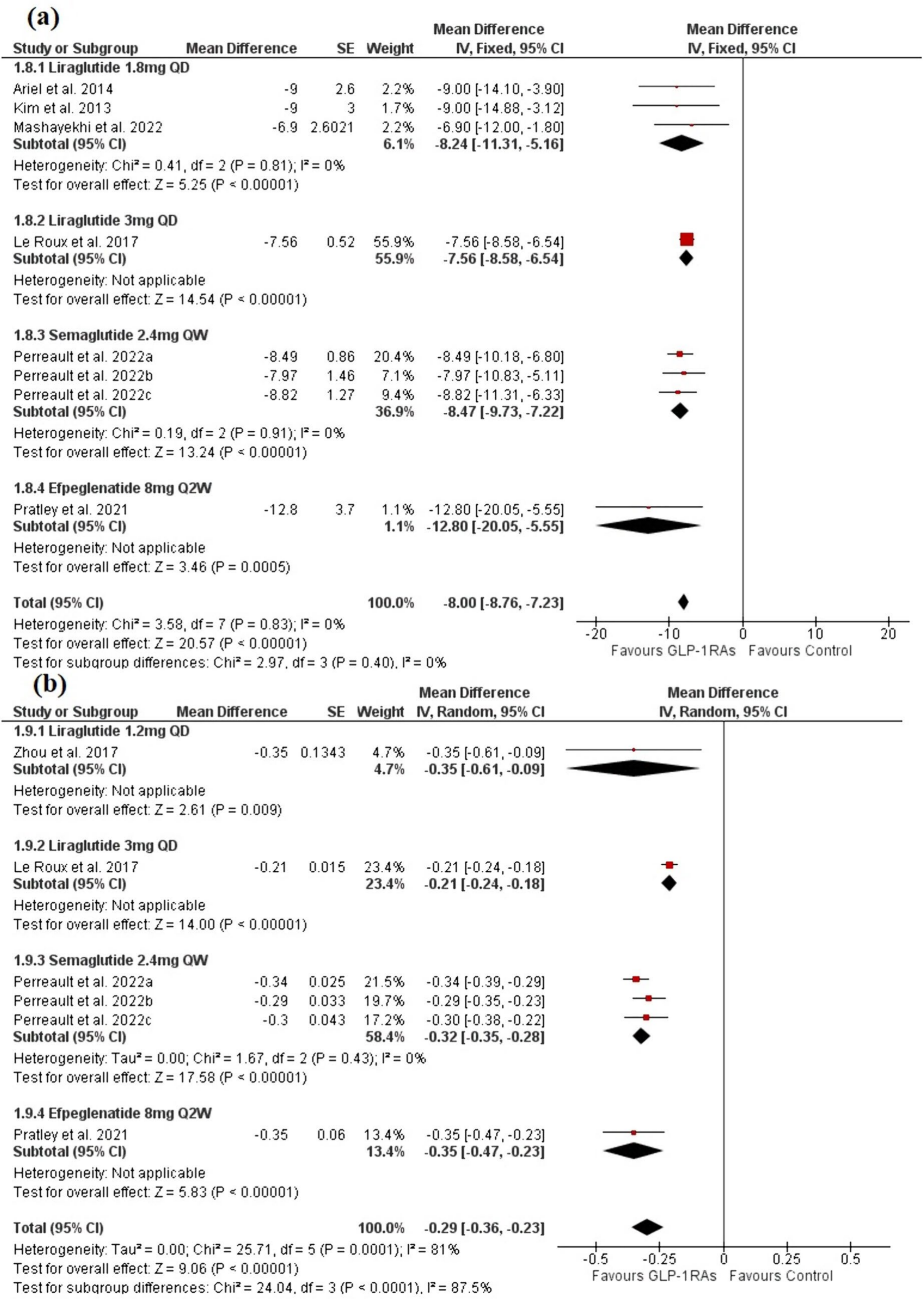
A forest plot shows the overall effect and subgroup analysis based on the type and dose of GLP-1RAs on **a** FPG and **b** HbA1c

The analysis included eight trials with 6 publications [26, 28, 39–41, 43] with a total of 2636 patients in the GLP-1RAs arm and 1297 patients in the control arm. Evidence with a high degree of certainty demonstrated that the GLP-1 group significantly decreased FPG compared to the control group [MD = -8.00, 95% CI (-8.76, -7.23), P < 0.00001]. The pooled analysis was homogenous (P > 0.1). The liraglutide 1.8mg and semaglutide 2.4mg QW significantly outperformed the control group in the subgroup analysis [MD = − 8.24, 95% CI (− 11.31, − 5.16), P < 0.00001], and [MD = − 8.47, 95% CI (− 9.73, − 7.22), P < 0.00001], respectively, and the pooled analysis was homogenous for both (P > 0.1) (Fig. 5a).

#### HbA1c (%)

The analysis included six trials with four publications [26, 28, 38, 39] with a total of 2568 patients in the GLP-1RAs arm and 1274 patients in the control arm. Evidence with a low degree of certainty demonstrated that the GLP-1 group significantly reduced HbA1c levels when compared to the control group [MD = − 0.29, 95% CI (− 0.36, − 0.23), P < 0.00001] (Fig. 5b). High heterogeneity was observed [I^2^ = 81%, P = 0.0001]. The heterogeneity was eliminated by excluding Le Roux et al. 2017 [26] [I^2^ = 0%, P = 0.74] without significant change in the pooled analysis [MD = − 0.32, 95% CI (− 0.35, − 0.29), P < 0.00001]. Semaglutide 2.4mg QW significantly outperformed the control group in the subgroup analysis [MD = − 0.32, 95% CI (− 0.35, − 0.28), P < 0.00001], and the pooled analysis was homogenous (P > 0.1) (Fig. 5b).

#### Weight loss (kg)

**Fig. 6:**
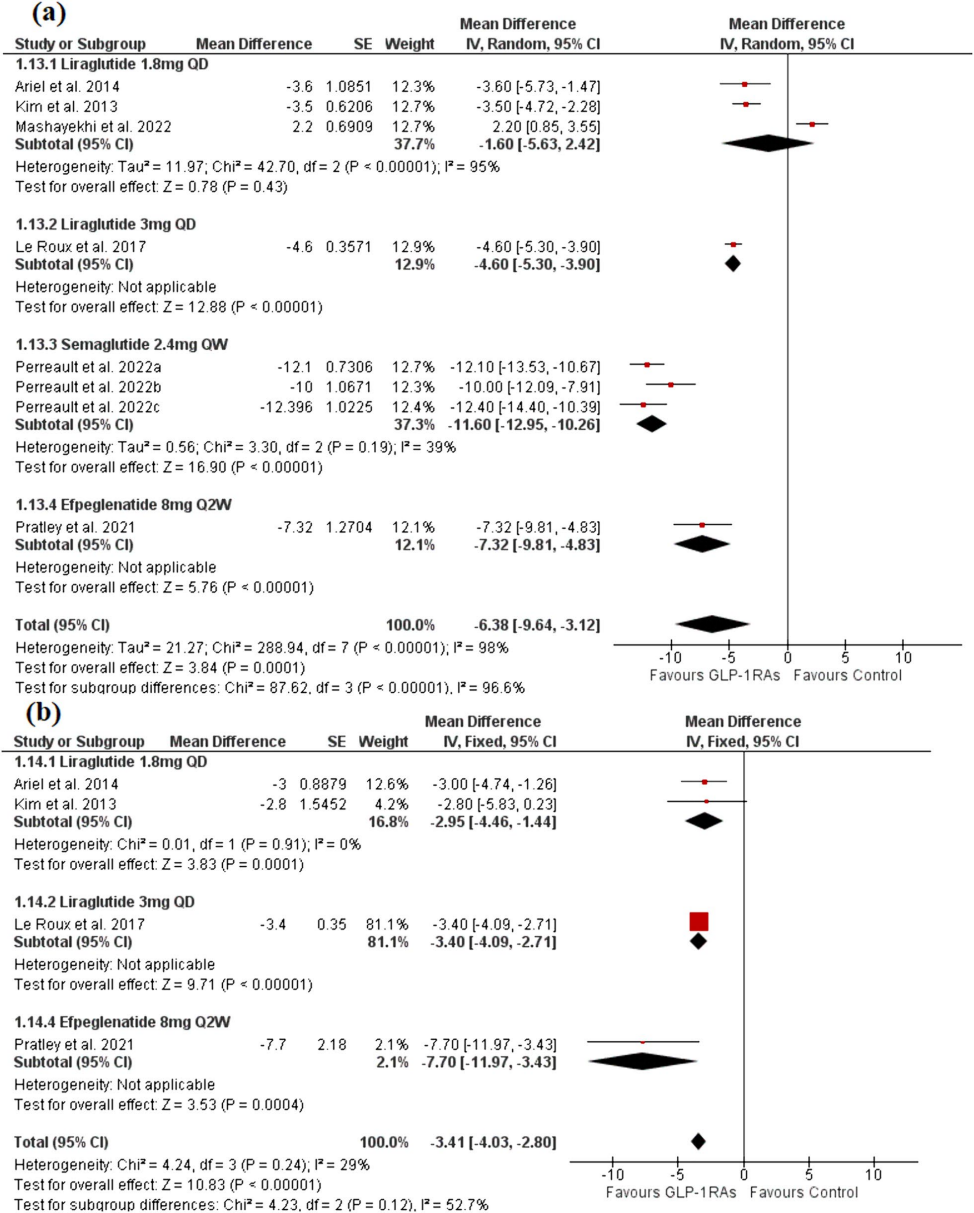
A forest plot shows the overall effect and subgroup analysis based on the type and dose of GLP-1RAs on **a** body weight and **b** waist circumference

The analysis included eight trials with six publications [26, 28, 39–41, 43] with a total of 2636 patients in the GLP-1RAs arm and 1297 patients in the control arm. Evidence with a very low degree of certainty demonstrated that the GLP-1 group significantly reduced body weight when compared to the control group [MD = − 6.38, 95% CI (− 9.64, − 3.12), P = 0.0001] (Fig. 6a). There was significant heterogeneity [I^2^ = 98%, P < 0.00001], which could not be resolved with the statistical analysis. Semaglutide 2.4mg QW significantly outperformed the control group in the subgroup analysis [MD = − 11.60, 95% CI (− 12.95, − 10.26), P < 0.00001] with the pooled analysis being homogenous (P > 0.1) while, liraglutide 1.8mg showed no significant difference compared to the control group in the subgroup analysis [MD = -1.60, 95% CI (− 5.63, 2.42), P = 0.43] (Fig. 6a). The pooled analysis was heterogeneous (P < 0.00001), which was resolved by the exclusion of Mashayekhi et al. 2022 (P > 0.01), after which the pooled analysis significantly favored the liraglutide 1.8mg group [MD = − 3.52, 95% CI (− 4.58, − 2.47), P = 0.43].

#### Waist circumference (cm)

The analysis included four trials [26, 39, 41, 43] with a total of 1543 patients in the GLP-1RAs arm and 822 patients in the control arm. Evidence with a moderate degree of certainty demonstrated that t00he GLP-1 group significantly reduced waist circumference compared to the control group [MD = − 3.41, 95% CI (− 4.03, − 2.80), P < 0.00001]. The pooled analysis was homogenous (P > 0.1). The liraglutide 1.8mg group significantly outperformed the control group in the subgroup analysis [MD = − 2.95, 95% CI (− 4.46, − 1.44), P = 0.0001]. The pooled analysis was homogenous (P > 0.1) (Fig. 6b).

#### Lipid profile (mg/dl)

**Fig. 7:**
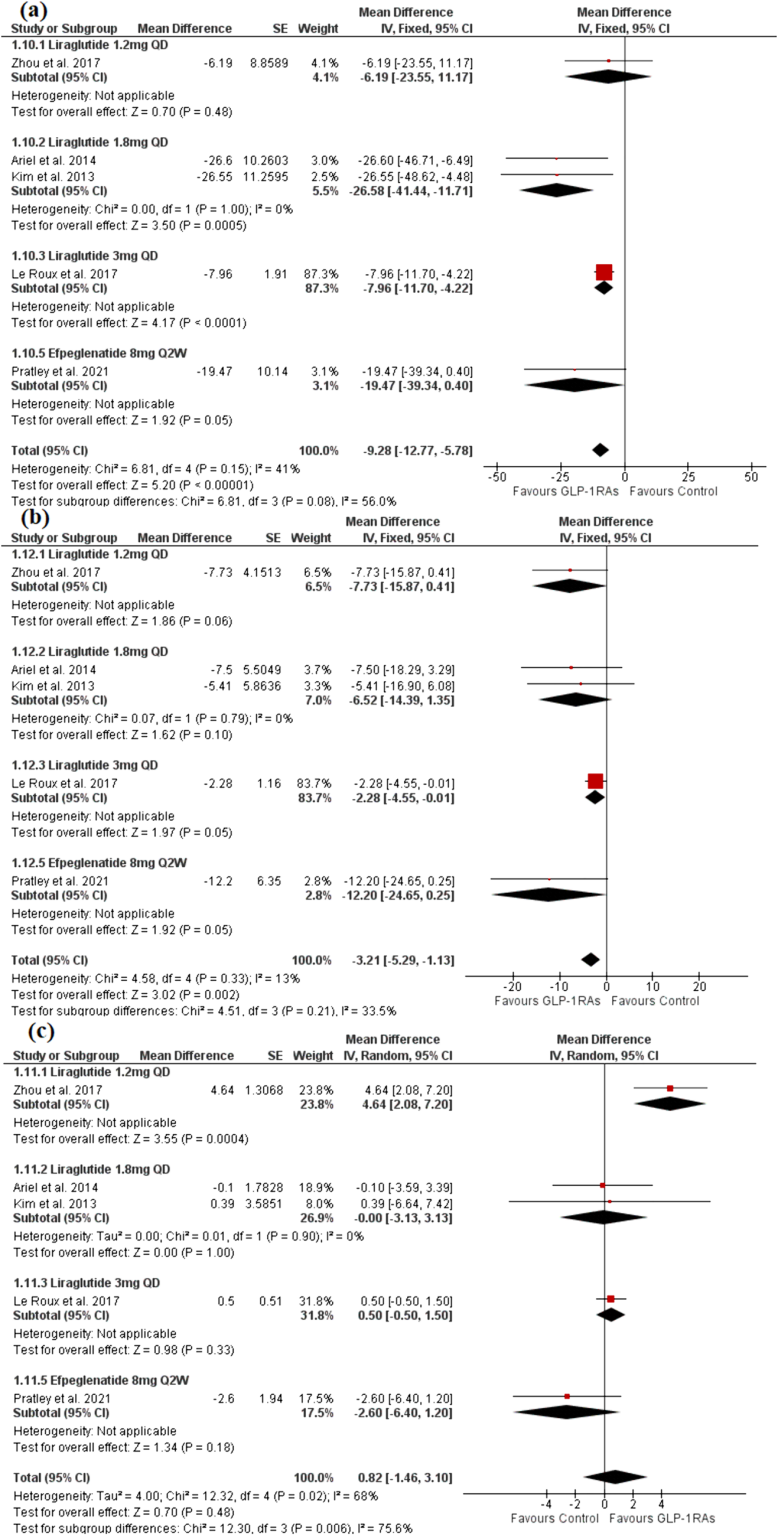
A forest plot shows the overall effect and subgroup analysis based on the type and dose of GLP-1RAs on **a** TG, **b** LDL, and **c** HDL

The analysis included five trials [26, 38, 39, 41, 43] with a total of 1564 patients in the GLP-1RAs arm and 843 patients in the control arm. The GLP-1 group significantly reduced TG and LDL compared to the control group [MD = − 9.28, 95% CI (− 12.77, − 5.78), P < 0.00001], [MD = − 3.21, 95% CI (− 5.29, − 1.13), P = 0.02] (Fig. 7a and b). The pooled analysis was homogeneous for both outcomes (P > 0.1). However, no significant difference was observed between the control group and the GLP-1 group regarding HDL [MD = 0.82, 95% CI (− 1.46, 3.10), P = 0.48] (Fig. 7c). The pooled analysis was heterogeneous (P = 0.02), which was resolved after the exclusion of Zhou et al. [38] (P > 0.1) without significant effect on the pooled analysis [MD = 0.27, 95% CI (− 0.65, 1.20), P = 0.56]. The evidence was of moderate, low, and very low degrees of certainty for TG, LDL, and HDL, respectively.

### Safety outcomes

**Fig. 8:**
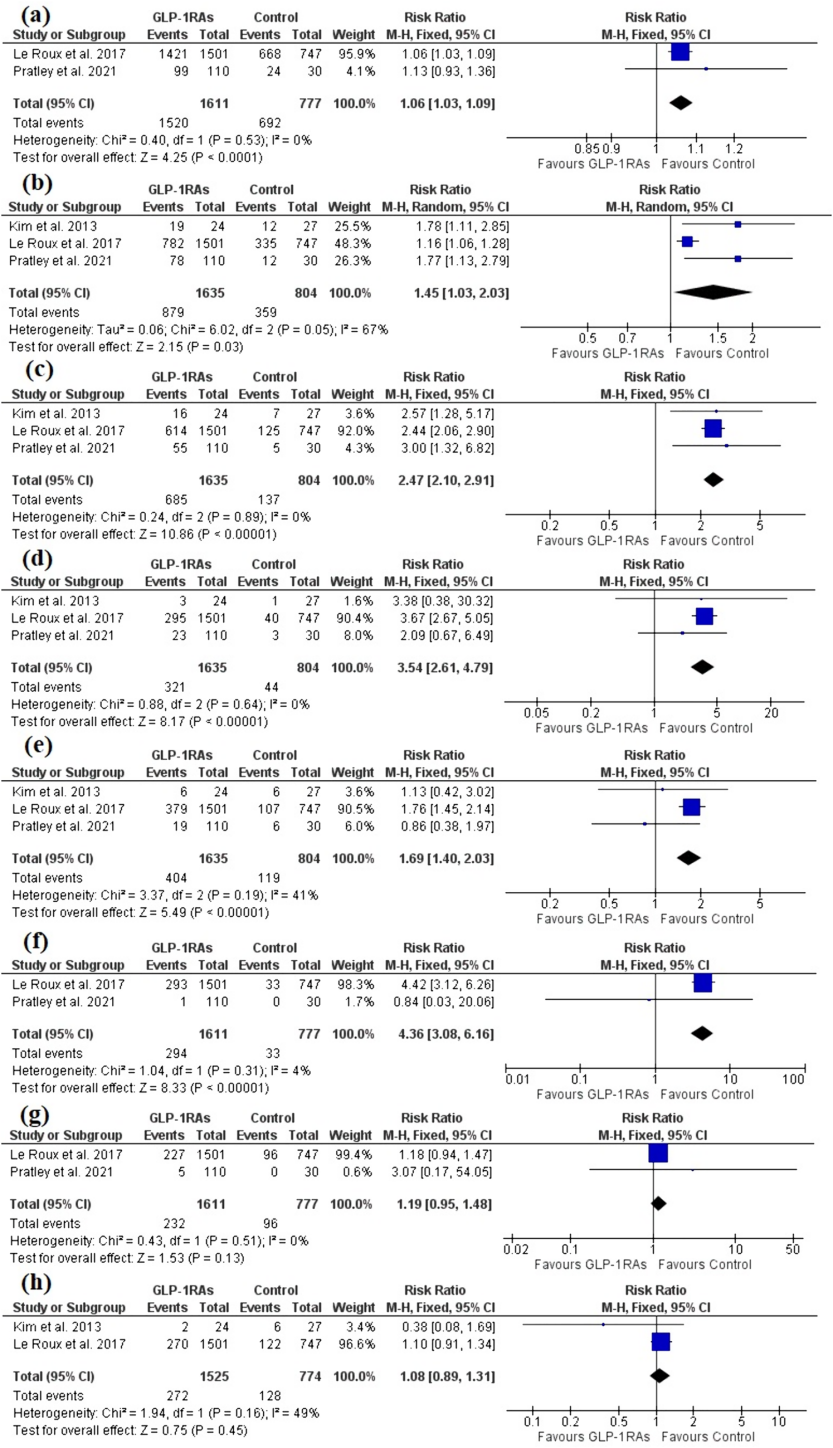
A forest plot shows the overall effect of GLP-1RAs on **a** Any adverse events, **b** Any gastrointestinal disorders, **c** nausea, **d** vomiting, **e** diarrhea, **f** hypoglycemia, **g** serious adverse event, **h** headache

Any gastrointestinal disorders, nausea, vomiting, and diarrhea adverse events were reported for prediabetic patients by three trials [26, 39, 43] with a total of 1635 patients in the GLP-1RAs arm and 804 patients in the control arm, and GLP-1RAs revealed a higher incidence of the aforementioned adverse events compared to the control [RR = 1.45, 95% CI (1.03, 2.03), P < 0.00001], [RR = 2.47, 95% CI (2.10, 2.91), P < 0.00001], [RR = 3.54, 95% CI (2.61, 4.79), P < 0.00001], [RR = 1.69, 95% CI (1.40, 2.03), P < 0.00001], respectively (Fig. 8b–e). All the pooled analyses were homogenous (P > 0.1) except for any gastrointestinal disorders, where significant heterogeneity was present [I^2^ = 67%, P = 0.05]. The heterogeneity was resolved by excluding Le Roux et al. 2017 [I^2^ = 0%, P = 0.99] without significant change in the pooled analysis [RR = 1.78, 95% CI (1.27, 2.48), P = 0.0008].

Any adverse events, hypoglycemia, and any serious adverse events were reported by two trials [26, 39] with a total of 1611 patients in the GLP-1RAs arm and 777 patients in the control arm, and GLP-1RAs revealed a higher incidence of Any adverse events and hypoglycemia [RR = 1.06, 95% CI (1.03, 1.09), P < 0.00001], [RR = 4.36, 95% CI (3.08, 6.16), P < 0.00001], while no significant difference was found regarding any serious adverse events [RR = 1.19, 95% CI (0.95, 1.48), P = 0.13] (Fig. 8a, f, and g). The pooled analyses were homogenous (P > 0.1).

GLP-1RAs did not show significant difference in terms of headache compared to the control [RR = 1.08, 95% CI (0.89, 1.31), P = 0.45], as reported by 2 studies [26, 43]. The pooled analysis was homogenous (P > 0.1) (Fig. 8h).

### Sensitivity analysis

Due to the differences in the included studies and patient characteristics, we performed a sensitivity analysis to see the robustness of our analysis. Lundkvist et al. [27] administered GLP-1RA combined with a sodium-glucose co-transporter 2 (SGLT2) inhibitor. Mashayekhi et al. [40] compared GLP-1RAs without lifestyle modification to the hypocaloric diet. Zhou et al. [38] included only patients aged 6 to 18 years old. As a result, these studies were excluded from the analysis, which revealed no significant change on the results.

### Trial sequential analysis

The TSA demonstrates that the cumulative z-curve had crossed the trial sequential monitoring boundary for the beneficial effect of GLP-1RAs therapy on the incidence of prediabetes reversion to normoglycemia (Figure S2) and the incidence of new-onset diabetes (Figure S3), and the actual cumulative sample size exceeded the required information size. This showed that the sample size was sufficient to draw firm conclusions regarding the beneficial effect of GLP-1RAs therapy on prediabetes reversion to normoglycemia and reducing the incidence of new-onset diabetes.

#### Publication bias

The funnel plots are shown in figures S4 to S12. No publication bias was observed in any of the efficacy outcomes as indicated by Egger’s (P > 0.05) and Begg’s (P > 0.05) tests, except for LDL, where there was significant publication bias as indicated by Egger’s test (P = 0.023). After applying the trim-and-fill method, it was revealed that after trimming three studies, no significant effect was noticed on the pooled estimate [MD = − 2.39, 95% CI (− 4.35, − 0.44)] (Figure S9).

## Discussion

The present systematic review and meta-analysis aim to evaluate the efficacy and safety of glucagon-like peptide-1 receptor agonists (GLP-1RAs) in the management of prediabetic patients. Our analysis included 11 trials comprising a total of 4,316 prediabetic patients, with follow-up durations ranging from 14 to 104 weeks. The findings of this study provide valuable insights into the potential benefits and limitations of GLP-1RAs in the context of prediabetes management. Our meta-analysis consistently demonstrates the positive effects of GLP-1RAs on prediabetes reversion to normoglycemia, prevention of overt diabetes, glycemic control, anthropometric parameters, and lipid profiles. These results collectively suggest that GLP-1RAs hold promise as a comprehensive therapeutic approach for prediabetic patients.

Our meta-analysis revealed that GLP-1RAs significantly increased the incidence of prediabetes reversion to normoglycemia in prediabetic patients compared to the control group. Prediabetes itself is associated with an increased risk of a spectrum of complications. Prediabetes increases the risk of cardiovascular events such as stroke, myocardial infarction, and cardiovascular cause-specific mortality [44]. Schlesingerc et al. [45] discovered that prediabetes was associated with an increased risk of all-cause mortality and incident cardiovascular events, coronary heart disease, stroke, heart failure, atrial fibrillation, chronic kidney disease, total cancer, total liver cancer, hepatocellular carcinoma, breast cancer, and all-cause dementia, with a moderate certainty of evidence. Furthermore, retinopathy, nephropathy, and peripheral neuropathy were all observed among individuals with prediabetes [46–48]. The mechanism underlying a higher likelihood of the aforementioned complications is proposed to be hypoglycemia-induced modifications in polyol, hexosamine, and protein kinase C (PKC) [49]. Our findings suggest that GLP-1RAs can effectively reverse prediabetes to normoglycemia as early as 14–24 weeks and last up to 68–104 weeks, potentially protecting against prediabetic complications.

Our analysis demonstrated a significant reduction in the incidence of new-onset diabetes in the GLP-1RAs group compared to the control group. Prediabetes has a yearly progression rate to diabetes of 10% [11]. The progression of prediabetic patients to diabetes adds an additional risk of complications, increasing the incidence of cardiovascular diseases such as hypertension, cardiac insufficiency, and coronary heart disease, as well as neurological diseases, renal failure, recurrent infections, retinopathy, and digestive disorders compared to non-diabetic patients [50]. Diabetes was responsible for 6.7 million deaths worldwide in 2021 [4]. Diabetes-related worldwide health spending was estimated to be 966 billion USD [51]. Therefore, pharmacological interventions such as GLP-1RAs can help reduce global economic burden, morbidity, and mortality by preventing the progression of prediabetes to diabetes. The protective effect of GLP-1RAs was observed after 48–56 weeks and lasted for 68–104 weeks; however, no significant effect was observed at 14–24 weeks, indicating that the preventive benefits of GLP-1RAs may take some time to manifest fully. The delayed onset of effect might be attributed to the gradual physiological impact of GLP-1RAs on insulin sensitivity, pancreatic function, and overall glycemic control [52, 53]. These findings are consistent with the natural progression of prediabetes to diabetes and highlight the importance of sustained intervention to achieve significant preventive effects.

GLP-1RAs demonstrated favorable effects on glycemic control, as evidenced by significant reductions in FPG and HbA1c levels compared to the control group. This was consistent across all the included studies. Lowering FPG and HbA1c levels contributes to reduced cardiovascular risk and improved overall health outcomes in diabetic and prediabetic patients [54, 55]. Furthermore, waist circumference has a stronger association with the risk of prediabetes [56]. Moreover, obese men and women have a sevenfold and 12-fold increase in the risk of developing diabetes, respectively [57]. Obesity increases adipose tissue fatty acid release, and visceral fat is related to increased circulating inflammatory cytokines and adhesion molecules, which have been linked to the development of insulin resistance and beta-cell dysfunction [58]. Our findings showed that GLP-1RAs had significant benefits for weight management and waist circumference reduction. This emphasizes their potential as comprehensive interventions for addressing prediabetes and its associated comorbidities and risk factors.

Although GLP-1RAs had favorable effects on body weight and waist circumference across all of the studies included, only Mashayekhi et al. [40] reported the opposite finding, which can be attributed to the fact that it was the only study that compared GLP-1RAs without lifestyle modification to lifestyle modification. This is consistent with earlier research in which lifestyle changes outperformed metformin in terms of bodyweight loss [11]. However, higher adherence to medication may offer comparable effect on the long term.

Our analysis also explored the impact of GLP-1RAs on lipid profiles, revealing notable effects on TG and LDL levels. For TG, this finding was consistent across all five studies that studied lipid profiles except for Zhou et al. [38], who studied liraglutide 1.2mg QD. For LDL, consistent lower values were observed across all the included studies except Rosenstock et al. [29], which could be attributed to the type and dose of GLP-1RAs or the small sample size. These findings suggest that beyond their glycemic benefits, GLP-1RAs may contribute to favorable changes in lipid metabolism, potentially lowering cardiovascular risk in prediabetic patients. The homogeneity observed in the pooled analyses for these outcomes indicates consistent effects across the studies. However, it's worth noting that there was no significant difference in high-density lipoprotein cholesterol (HDL-C) levels between the GLP-1RA group and the control group. These findings warrant further investigation into the underlying mechanisms and the possible dose-dependent or GLP-1RAs type-specific effect on the LDL. While these lipid-lowering effects are encouraging, the clinical implications need to be considered in the context of the broader cardiovascular risk profile of prediabetic patients. The observed reductions in TG and LDL-C levels, when coupled with improvements in glycemic control and other anthropometric measures, could collectively contribute to a more favorable cardiovascular risk profile in this population.

The safety analysis revealed that GLP-1RAs were associated with a higher incidence of gastrointestinal adverse events, such as nausea, vomiting, and diarrhea, compared to the control group. These side effects are consistent with the known gastrointestinal effects of GLP-1RAs, which are generally considered tolerable and transient in nature. These events often occur due to the effects of GLP-1RAs on gastric emptying and satiety. Although they may lead to treatment discontinuation in some cases, appropriate patient education and management strategies can mitigate their impact and improve treatment adherence. It is essential for clinicians to be aware of these potential adverse events and to counsel patients accordingly to ensure adherence to treatment. Importantly, our analysis did not find a significant increase in serious adverse events associated with GLP-1RA use in prediabetic patients. This finding is reassuring and suggests that GLP-1RAs can be tolerable for use in this population with an overall favorable benefit-risk profile.

According to the American Diabetes Association, the current recommended treatment for prediabetes is metformin along with lifestyle modification [59]. We revealed that GLP-1RAs combined with lifestyle modification resulted in better management than lifestyle modification alone. Our findings guide future guidelines to include GLP-1RAs as an addition to lifestyle modification instead of metformin in the management of prediabetic individuals who are intolerant to metformin. Further comparative studies are needed to identify the most effective medication along with lifestyle modification.

### Strengths and limitations

A comprehensive, large-scale search was conducted in the present study to retrieve all potentially relevant studies. Furthermore, the majority of the studies included were of high quality, and no publication bias was found. The majority of the pooled effects of the outcomes were homogenous, adding to the robustness of the findings. We performed a sensitivity analysis by omitting studies with different characteristics to evaluate how robust our conclusions are, and the results showed no significant changes. However, there are some limits. Some of the included studies did not mention if patients did not take any therapies that could directly or indirectly influence circulating glucose values. However, the majority reported that they excluded patients receiving weight loss medications or medications affecting carbohydrate metabolism. Our primary outcome had a high degree of heterogeneity that could only be partially resolved. However, subgroup analysis revealed that the duration of the treatment could explain this. Furthermore, due to limited data, we were unable to draw firm conclusions about the effects of different doses and types of GLP-1RAs.

## Conclusions

GLP-1RAs added to lifestyle modification achieved better effects on prediabetes reversion to normoglycemia, prevention of overt diabetes, glycemic control, anthropometric parameters, and lipid profiles, with a tolerable safety profile without significant serious adverse events. Future guidelines may propose the use of GLP-1RAs along with lifestyle modification to improve prediabetic patient management and therapy adherence. In addition, for people with metformin intolerance, GLP-1RAs may be a feasible alternative therapy. Future studies are needed to establish the most effective medication that can be used in combination with lifestyle modification.

### Supplementary Information

Supplementary Material 1.

## Data Availability

All data analyzed during this study are included in this published article or listed in references.

## Abbreviations

ADA: American Diabetes Association
AEs: Adverse events
BMI: Body mass index
CI: Confidence intervals
FPG: Fasting plasma glucose
GLP-1RAs: Glucagon-like peptide 1 receptor agonists
HbA1c: Glycated hemoglobin
HDL: High-density lipoprotein
IFG: Impaired fasting glucose
IGT: Impaired glucose tolerance
LDL: Low-density lipoprotein
MD: Mean difference
PRISMA: Preferred Reporting Items for Systematic Reviews and Meta-Analyses
RCTs: Randomized controlled trials
ROB: Risk of bias
RR: Risk ratio
SD: Standard deviation
T2DM: Type 2 diabetes mellitus
TG: Triglyceride
