# Supplementary material for: Efficacy and safety of glucagon-like peptide-1 receptor agonists on prediabetes: a systematic review and meta-analysis of randomized controlled trials
Source: Diabetol Metab Syndr. 2024 Jun 14;16:129. doi: 10.1186/s13098-024-01371-3 (PMC11177512; doi:10.1186/s13098-024-01371-3)
Supplement: Supplementary file 1 — Supplementary Material 1. [file 13098_2024_1371_MOESM1_ESM.docx]

**Contents:**

**Table S1.** Full search strategy for each database.

**Table S2.** The inclusion and exclusion criteria of the included studies.

**Table S3.** Quality assessment of the outcomes using GRADE criteria

**Figure S1.** The effect of the different doses of the different GLP-1RAs on the incidence of prediabetes reversion to normoglycemia.

**Figure S2.** Trial sequential analysis of the incidence of prediabetes reversion to normoglycemia.

**Figure S3.** Trial sequential analysis of the incidence of new-onset diabetes.

**Figure S4.** Funnel plot of the publication bias of prediabetes reversion to normoglycemia.

**Figure S5.** Funnel plot of the publication bias of new-onset diabetes.

**Figure S6.** Funnel plot of the publication bias of FPG.

**Figure S7.** Funnel plot of the publication bias of HbA1c.

**Figure S8.** Funnel plot of the publication bias of triglycerides.

**Figure S9.** Funnel plot of the publication bias of LDL.

**Figure S10.** Funnel plot of the publication bias of HDL.

**Figure S11.** Funnel plot of the publication bias of body weight change.

**Figure S12.** Funnel plot of the publication bias of waist circumference change.

| Database | Search Terms | Search Field | Search Results |
| --- | --- | --- | --- |
| PubMed | (("Glucagon Like Peptides" OR "Glucagon-Like Polypeptide" OR "Glucagon Like Polypeptide" OR "Polypeptide, Glucagon-Like" OR "Glucagon-Like Polypeptides" OR "Glucagon Like Polypeptides" OR "Glucagon-Like Peptide" OR "Glucagon Like Peptide" OR "Peptide, Glucagon-Like" OR enteroglucagons OR enteroglucagon OR "Gut Glucagon" OR "Glucagon, Gut" OR "Glucagon Like Peptide 1" OR glp-1 OR "GLP 1" OR "Glucagon-Like Peptide-1" OR "Glucagon Like Peptide 2" OR "Proglucagon (126-158)" OR "Glucagon-Like Peptide-2 (1-33)" OR "Glucagon-Like Peptide-1 Receptor Agonist" OR "Dual GIP and GLP-1 Receptor Agonist" OR Exenatide OR Bydureon OR "ITCA 650" OR "AC 2993 LAR" OR "Exendin-4" OR "Ex4 Peptide" OR "Peptide, Ex4" OR "Exendin 4" OR byetta OR "AC 2993" OR semaglutide OR rybelsus OR Ozempic OR dulaglutide OR "LY 2189265" OR "LY-2189265" OR ly2189265 OR trulicity OR eperzan OR tanzeum OR albiglutide OR efpeglenatide OR "L-Proline, 1-(3-(2-(3-(((5S)-5-amino-5-carboxypentyl)amino)propoxy)ethoxy)propyl)" OR lixisenatide OR "DES-38-proline-exendine-4 (Heloderma suspectum)-(1-39)-peptidylpenta-l-lysyl-l-lysinamide" OR adlyxin OR "AQVE-10010" OR "ZP10A peptide" OR "ZP 10" OR "ZP-10" OR lyxumia OR "AVE 010" OR "AVE-010" OR "AVE 0010" OR ave0010 OR ave-0010 OR liraglutide OR victoza OR saxenda OR "NN 221" OR "2211, NN" OR nn2211 OR nn-2211 OR taspoglutide OR tirzepatide OR ly3298176) AND (Obesity OR Obes* OR "Prediabetic States" OR "State, Prediabetic" OR "States, Prediabetic" OR Prediabetes OR Overweight) AND (trial)) | All Fields | 2144 |
| Cochrane | (("Glucagon Like Peptides" OR "Glucagon-Like Polypeptide" OR "Glucagon Like Polypeptide" OR "Polypeptide, Glucagon-Like" OR "Glucagon-Like Polypeptides" OR "Glucagon Like Polypeptides" OR "Glucagon-Like Peptide" OR "Glucagon Like Peptide" OR "Peptide, Glucagon-Like" OR enteroglucagons OR enteroglucagon OR "Gut Glucagon" OR "Glucagon, Gut" OR "Glucagon Like Peptide 1" OR glp-1 OR "GLP 1" OR "Glucagon-Like Peptide-1" OR "Glucagon Like Peptide 2" OR "Proglucagon (126-158)" OR "Glucagon-Like Peptide-2 (1-33)" OR "Glucagon-Like Peptide-1 Receptor Agonist" OR "Dual GIP and GLP-1 Receptor Agonist" OR Exenatide OR Bydureon OR "ITCA 650" OR "AC 2993 LAR" OR "Exendin-4" OR "Ex4 Peptide" OR "Peptide, Ex4" OR "Exendin 4" OR byetta OR "AC 2993" OR semaglutide OR rybelsus OR Ozempic OR dulaglutide OR "LY 2189265" OR "LY-2189265" OR ly2189265 OR trulicity OR eperzan OR tanzeum OR albiglutide OR efpeglenatide OR "L-Proline, 1-(3-(2-(3-(((5S)-5-amino-5-carboxypentyl)amino)propoxy)ethoxy)propyl)" OR lixisenatide OR "DES-38-proline-exendine-4 (Heloderma suspectum)-(1-39)-peptidylpenta-l-lysyl-l-lysinamide" OR adlyxin OR "AQVE-10010" OR "ZP10A peptide" OR "ZP 10" OR "ZP-10" OR lyxumia OR "AVE 010" OR "AVE-010" OR "AVE 0010" OR ave0010 OR ave-0010 OR liraglutide OR victoza OR saxenda OR "NN 221" OR "2211, NN" OR nn2211 OR nn-2211 OR taspoglutide OR tirzepatide OR ly3298176) AND (Obesity OR Obes* OR "Prediabetic States" OR "State, Prediabetic" OR "States, Prediabetic" OR Prediabetes OR Overweight) AND (trial)) | All Fields | 2396 |
| WOS | (("Glucagon Like Peptides" OR "Glucagon-Like Polypeptide" OR "Glucagon Like Polypeptide" OR "Polypeptide, Glucagon-Like" OR "Glucagon-Like Polypeptides" OR "Glucagon Like Polypeptides" OR "Glucagon-Like Peptide" OR "Glucagon Like Peptide" OR "Peptide, Glucagon-Like" OR enteroglucagons OR enteroglucagon OR "Gut Glucagon" OR "Glucagon, Gut" OR "Glucagon Like Peptide 1" OR glp-1 OR "GLP 1" OR "Glucagon-Like Peptide-1" OR "Glucagon Like Peptide 2" OR "Proglucagon (126-158)" OR "Glucagon-Like Peptide-2 (1-33)" OR "Glucagon-Like Peptide-1 Receptor Agonist" OR "Dual GIP and GLP-1 Receptor Agonist" OR Exenatide OR Bydureon OR "ITCA 650" OR "AC 2993 LAR" OR "Exendin-4" OR "Ex4 Peptide" OR "Peptide, Ex4" OR "Exendin 4" OR byetta OR "AC 2993" OR semaglutide OR rybelsus OR Ozempic OR dulaglutide OR "LY 2189265" OR "LY-2189265" OR ly2189265 OR trulicity OR eperzan OR tanzeum OR albiglutide OR efpeglenatide OR "L-Proline, 1-(3-(2-(3-(((5S)-5-amino-5-carboxypentyl)amino)propoxy)ethoxy)propyl)" OR lixisenatide OR "DES-38-proline-exendine-4 (Heloderma suspectum)-(1-39)-peptidylpenta-l-lysyl-l-lysinamide" OR adlyxin OR "AQVE-10010" OR "ZP10A peptide" OR "ZP 10" OR "ZP-10" OR lyxumia OR "AVE 010" OR "AVE-010" OR "AVE 0010" OR ave0010 OR ave-0010 OR liraglutide OR victoza OR saxenda OR "NN 221" OR "2211, NN" OR nn2211 OR nn-2211 OR taspoglutide OR tirzepatide OR ly3298176) AND (Obesity OR Obes* OR "Prediabetic States" OR "State, Prediabetic" OR "States, Prediabetic" OR Prediabetes OR Overweight) AND (trial)) | All Fields | 5983 |
| SCOPUS | TITLE-ABS-KEY((("Glucagon Like Peptides" OR "Glucagon-Like Polypeptide" OR "Glucagon Like Polypeptide" OR "Polypeptide, Glucagon-Like" OR "Glucagon-Like Polypeptides" OR "Glucagon Like Polypeptides" OR "Glucagon-Like Peptide" OR "Glucagon Like Peptide" OR "Peptide, Glucagon-Like" OR enteroglucagons OR enteroglucagon OR "Gut Glucagon" OR "Glucagon, Gut" OR "Glucagon Like Peptide 1" OR glp-1 OR "GLP 1" OR "Glucagon-Like Peptide-1" OR "Glucagon Like Peptide 2" OR "Proglucagon (126-158)" OR "Glucagon-Like Peptide-2 (1-33)" OR "Glucagon-Like Peptide-1 Receptor Agonist" OR "Dual GIP and GLP-1 Receptor Agonist" OR Exenatide OR Bydureon OR "ITCA 650" OR "AC 2993 LAR" OR "Exendin-4" OR "Ex4 Peptide" OR "Peptide, Ex4" OR "Exendin 4" OR byetta OR "AC 2993" OR semaglutide OR rybelsus OR Ozempic OR dulaglutide OR "LY 2189265" OR "LY-2189265" OR ly2189265 OR trulicity OR eperzan OR tanzeum OR albiglutide OR efpeglenatide OR "L-Proline, 1-(3-(2-(3-(((5S)-5-amino-5-carboxypentyl)amino)propoxy)ethoxy)propyl)" OR lixisenatide OR "DES-38-proline-exendine-4 (Heloderma suspectum)-(1-39)-peptidylpenta-l-lysyl-l-lysinamide" OR adlyxin OR "AQVE-10010" OR "ZP10A peptide" OR "ZP 10" OR "ZP-10" OR lyxumia OR "AVE 010" OR "AVE-010" OR "AVE 0010" OR ave0010 OR ave-0010 OR liraglutide OR victoza OR saxenda OR "NN 221" OR "2211, NN" OR nn2211 OR nn-2211 OR taspoglutide OR tirzepatide OR ly3298176) AND (Obesity OR Obes* OR "Prediabetic States" OR "State, Prediabetic" OR "States, Prediabetic" OR Prediabetes OR Overweight) AND (trial))) | Title, Abstract, Keywords | 2935 |

**Table S1**. Full search strategy for each database.

**Table S2.** The inclusion and exclusion criteria of the included studies

| Study ID | Inclusion criteria | Exclusion criteria |
| --- | --- | --- |
| Ariel et al. 2014 | Age ≥ 40 years, overweight with prediabetes, in good general health, without known disease | T2DM, kidney or liver disease, any medications that affect lipoprotein or carbohydrate metabolism or promote weight loss, had a history of gallstones, pancreatitis, medullary carcinoma, family history of medullary carcinoma or multiple endocrine neoplasia type 2. |
| Rosenstock et al. 2010 | Non diabetic, obese (BMI ≥ 30 kg/m²) with or without prediabetes | T2DM, previous use of glucose-lowering medications for more than 3 months, unstable body weight |
| Mashayekhi et al. 2022 | Age = 18 to 65 years, obese (BMI ≥ 30 kg/m²) with prediabetes | Type 1 or 2 diabetes, resistant hypertension, history of pancreatitis, significant cardiovascular disease, asthma with regular inhaler use, and impaired kidney or liver function |
| Pratley et al. 2021 | Obesity/overweight (BMI ≥30 kg/m² or ≥27 kg/m²) with prediabetes (FPG < 126mg/dL) with comorbidities | BMI > 42kg/m2, drug-induced (iatrogenic) obesity, known diabetes, or HbA1c >6.5% (48mmol/mol). |
| Kim et al. 2013 | Age = 40 to 70 years, overweight/obesity (BMI = 27 to 40 kg/m²) with prediabetes and stable weight (< 5% reported change) in the previous 3 months. | T2DM, use of medications that can affect carbohydrate metabolism or promote weight loss, gallstones, history of pancreatitis, medullary carcinoma, family history of medullary carcinoma or multiple endocrine neoplasia type 2, and known cardiac, liver, or kidney disease. |
| Lundkvist et al. 2017 | Age = 18 to 70 years, non-diabetic, obese (BMI = 30 to 45 kg/m²) with or without prediabetes | T2DM |
| Zhou et al. 2017 | Children (Age = 6 to 18 years), overweight/obese with prediabetes | Any children with genetic metabolism, endocrine disease, kidney disease high blood pressure and high blood lipid profile |
| Astrup et al. 2009 | Age = 18 to 65 years, non-diabetic, obese (BMI = 30–40 kg/m²) with or without prediabetes with stable bodyweight (<5% reported change during the previous 3 months) and fasting plasma glucose of less than 7 mmol/L. | Type 1 or 2 diabetes, obesity induced by drug treatment, use of approved weight-lowering pharmacotherapy or participation in a clinical weight control study within the previous 3 months, previous surgical obesity treatment, and major medical conditions |
| Astrup et al. 2012 | Age = 18 to 65 years, non-diabetic, obese (BMI = 30–40 kg/m²) with or without prediabetes with stable bodyweight (<5% reported change during the previous 3 months) and fasting plasma glucose of less than 7 mmol/L. | Type 1 or 2 diabetes, obesity induced by drug treatment, use of approved weight-lowering pharmacotherapy or participation in a clinical weight control study within the previous 3 months, previous surgical obesity treatment, and major medical conditions |
| Pi-Sunyer et al. 2015 | Age ≥ 18 years, non-diabetic, obese/overweight (BMI ≥ 30.0 kg/m2 or ≥27.0 kg/m2) with or without prediabetes with treated or untreated comorbid dyslipidemia and/or hypertension, stable body weight (< 5 kg self-reported change during the previous 3 months), and Preceding failed dietary effort | Type 1 or 2 diabetes, the use of medications that cause clinically significant weight gain or loss, previous bariatric surgery, a history of pancreatitis, a history of major depressive or other severe psychiatric disorders, and a family or personal history of multiple endocrine neoplasia type 2 or familial medullary thyroid carcinoma. |
| Le Roux et al. 2017 | Age ≥ 18 years, Overweight/obesity (BMI ≥ 30.0 kg/m2 or ≥27.0 kg/m2) with prediabetes with treated or untreated comorbid dyslipidemia and/or hypertension, stable body weight (< 5 kg self-reported change during the previous 3 months), and Preceding failed dietary effort | Type 1 or 2 diabetes, the use of medications that cause clinically significant weight gain or loss, previous bariatric surgery, a history of pancreatitis, a history of major depressive or other severe psychiatric disorders, and a family or personal history of multiple endocrine neoplasia type 2 or familial medullary thyroid carcinoma. |
| Perreault et al. 2022a | Age ≥18 years, non-diabetic, overweight/obese (BMI ≥ 30.0 kg/m² or ≥27.0 kg/m²) with or without prediabetes with one or more treated or untreated weight-related coexisting conditions (i.e., hypertension, dyslipidemia, obstructive sleep apnea, or cardiovascular disease), and History of at least one self-reported unsuccessful dietary effort to lose body weight. | Diabetes, HbA1c = 48 mmol/L (6.5%) or greater, a history of chronic pancreatitis, acute pancreatitis within 180 days before enrollment, previous surgical obesity treatment, and use of anti-obesity medication within 90 days before enrollment. |
| Perreault et al. 2022b | Age ≥18 years, non-diabetic, overweight/obese (BMI ≥ 30.0 kg/m² or ≥27.0 kg/m²) with or without prediabetes with one or more treated or untreated weight-related coexisting conditions (i.e., hypertension, dyslipidemia, obstructive sleep apnea, or cardiovascular disease), and History of at least one self-reported unsuccessful dietary effort to lose body weight. | Diabetes, HbA1c = 48 mmol/L (6.5%) or greater, a history of chronic pancreatitis, acute pancreatitis within 180 days before enrollment, previous surgical obesity treatment, and use of anti-obesity medication within 90 days before enrollment. |
| Perreault et al. 2022c | Age ≥18 years, non-diabetic, overweight/obese (BMI ≥ 30.0 kg/m² or ≥27.0 kg/m²) with or without prediabetes with one or more treated or untreated weight-related coexisting conditions (i.e., hypertension, dyslipidemia, obstructive sleep apnea, or cardiovascular disease), and History of at least one self-reported unsuccessful dietary effort to lose body weight. All patients received subcutaneous semaglutide for a 20-week run-in period | Diabetes, HbA1c = 48 mmol/L (6.5%) or greater, a history of chronic pancreatitis, acute pancreatitis within 180 days before enrollment, previous surgical obesity treatment, and use of anti-obesity medication within 90 days before enrollment. |

**Table S3.** Quality assessment of the outcomes using GRADE criteria.

| **Certainty assessment** | | | | | | | **№ of patients** | | **Effect** | | **Certainty** | **Importance** |
| --- | --- | --- | --- | --- | --- | --- | --- | --- | --- | --- | --- | --- |
| **№ of studies** | **Study design** | **Risk of bias** | **Inconsistency** | **Indirectness** | **Imprecision** | **Other considerations** | **[intervention]** | **[comparison]** | **Relative (95% CI)** | **Absolute (95% CI)** |  |  |
| **The incidence of prediabetes reversion to normoglycemia** | | | | | | | | | | | | |
| 9 | randomised trials | not serious | serious^a^ | serious^b^ | not serious | none | 2000/2817 (71.0%) | 550/1348 (40.8%) | **RR 1.76** (1.45 to 2.13) | **310 more per 1,000** (from 184 more to 461 more) | ⨁⨁◯◯ Low |  |
| **The incidence of new-onset diabetes** | | | | | | | | | | | | |
| 6 | randomised trials | not serious | not serious | serious^b^ | not serious | none | 31/2557 (1.2%) | 58/1258 (4.6%) | **RR 0.28** (0.19 to 0.43) | **33 fewer per 1,000** (from 37 fewer to 26 fewer) | ⨁⨁⨁◯ Moderate |  |
| **FPG** | | | | | | | | | | | | |
| 6 | randomised trials | not serious | not serious | serious^c^ | not serious | strong association | 2636 | 1297 | - | MD **8 mg/dl lower** (8.76 lower to 7.23 lower) | ⨁⨁⨁⨁ High |  |
| **HbA1c** | | | | | | | | | | | | |
| 6 | randomised trials | not serious | serious^d^ | serious^c^ | not serious | none | 2568 | 1274 | - | MD **0.29 % lower** (0.36 lower to 0.23 lower) | ⨁⨁◯◯ Low |  |
| **Weight loss** | | | | | | | | | | | | |
| 8 | randomised trials | not serious | very serious^e^ | serious^c^ | serious^f^ | none | 2636 | 1297 | - | MD **6.38 kg lower** (9.64 lower to 3.12 lower) | ⨁◯◯◯ Very low |  |
| **Waist circumference** | | | | | | | | | | | | |
| 4 | randomised trials | not serious | not serious | serious^c^ | not serious | none | 1543 | 822 | - | MD **3.41 cm lower** (4.03 lower to 2.8 lower) | ⨁⨁⨁◯ Moderate |  |
| **TG** | | | | | | | | | | | | |
| 5 | randomised trials | not serious | not serious | serious^c^ | not serious | none | 1564 | 843 | - | MD **9.28 mg/dl lower** (12.77 lower to 5.78 lower) | ⨁⨁⨁◯ Moderate |  |
| **LDL** | | | | | | | | | | | | |
| 5 | randomised trials | not serious | not serious | serious^c^ | not serious | publication bias strongly suspected^g^ | 1564 | 843 | - | MD **3.21 mg/dl lower** (5.29 lower to 1.13 lower) | ⨁⨁◯◯ Low |  |
| **HDL** | | | | | | | | | | | | |
| 5 | randomised trials | not serious | serious^d^ | serious^c^ | serious^f^ | none | 1564 | 843 | - | MD **0.82 mg/dl higher** (1.46 lower to 3.1 higher) | ⨁◯◯◯ Very low |  |

**CI:** confidence interval; **MD:** mean difference; **RR:** risk ratio

#### Explanations

a. High heterogeneity, which could be partially explained by the different follow-up durations

b. Different doses, durations of follow-up, and GLP-1RAs agents. However, subgroup analyses for these variables were conducted

c. Different doses and GLP-1RA agents. However, subgroup analyses for these variables were conducted

d. High heterogeneity, which could be partially explained by the different GLP-11RA agents

e. Very high heterogeneity

f. Confidence intervals do not overlap

g. Egger's test (P = 0.023). However, After applying the trim-and-fill method, no significant effect was noticed on the pooled estimate


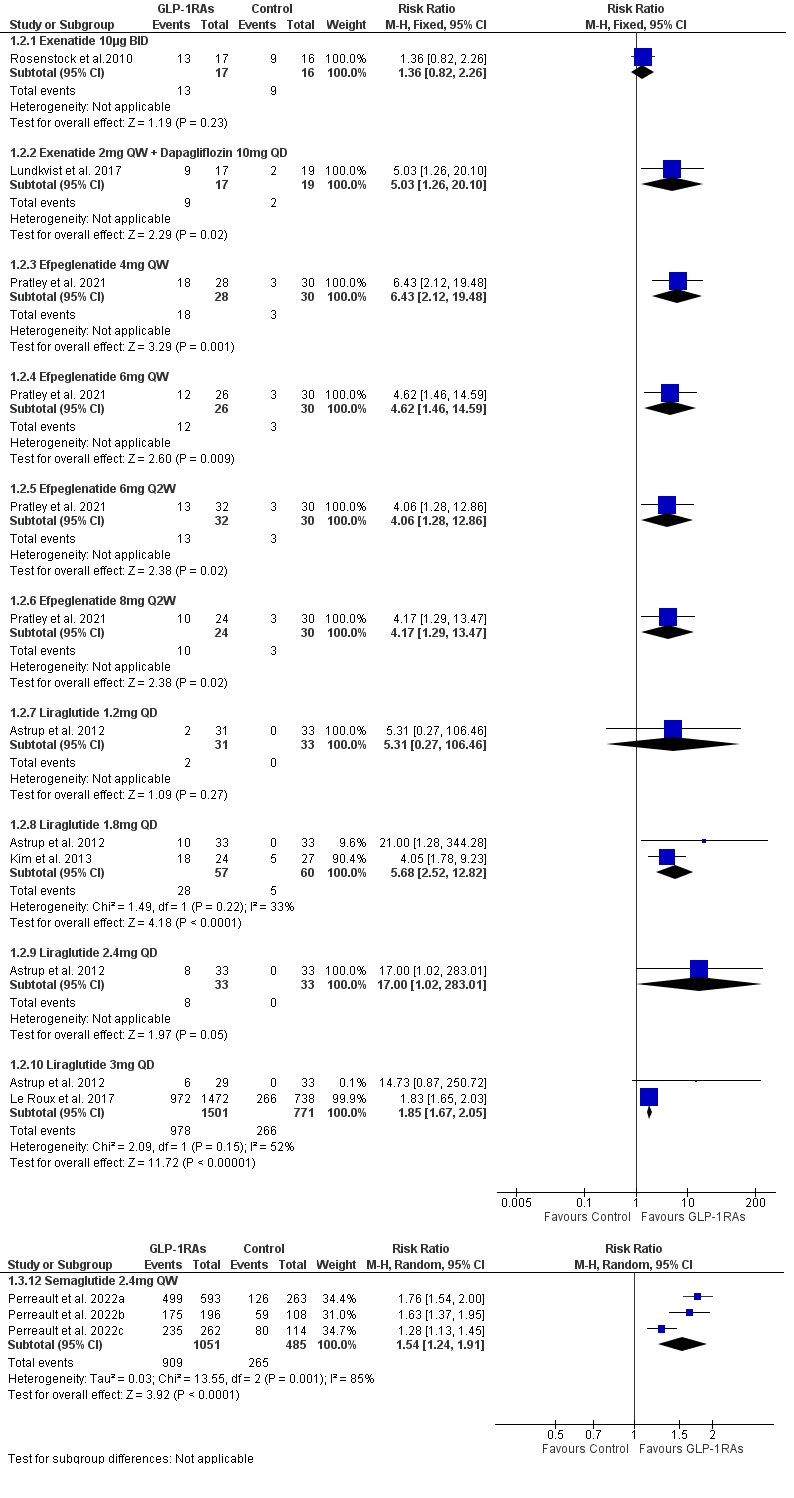


**Figure S1.** The effect of the different doses of the different GLP-1RAs on the incidence of prediabetes reversion to normoglycemia.

**
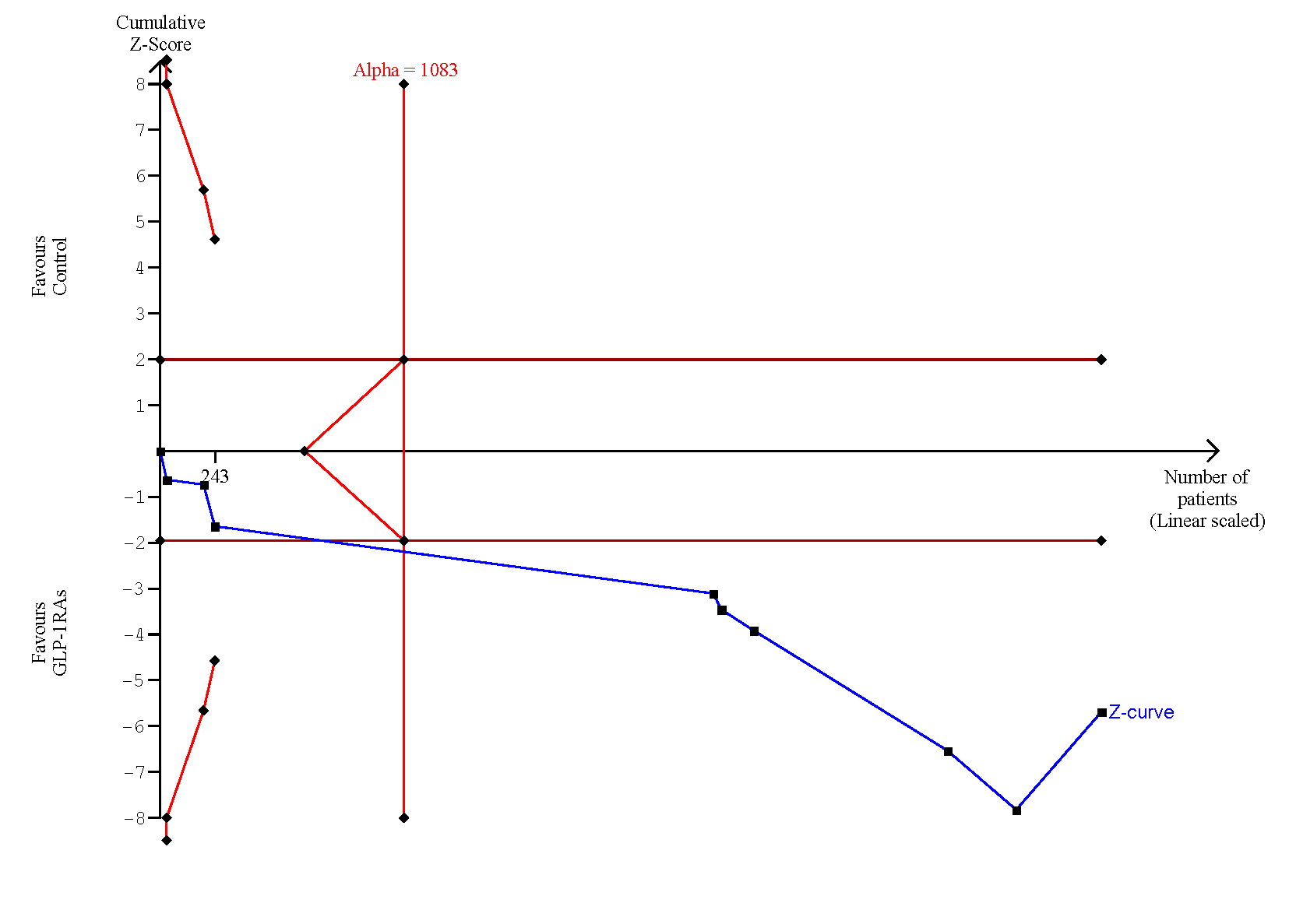
Figure S2.** Trial sequential analysis of the incidence of prediabetes reversion to normoglycemia.

**
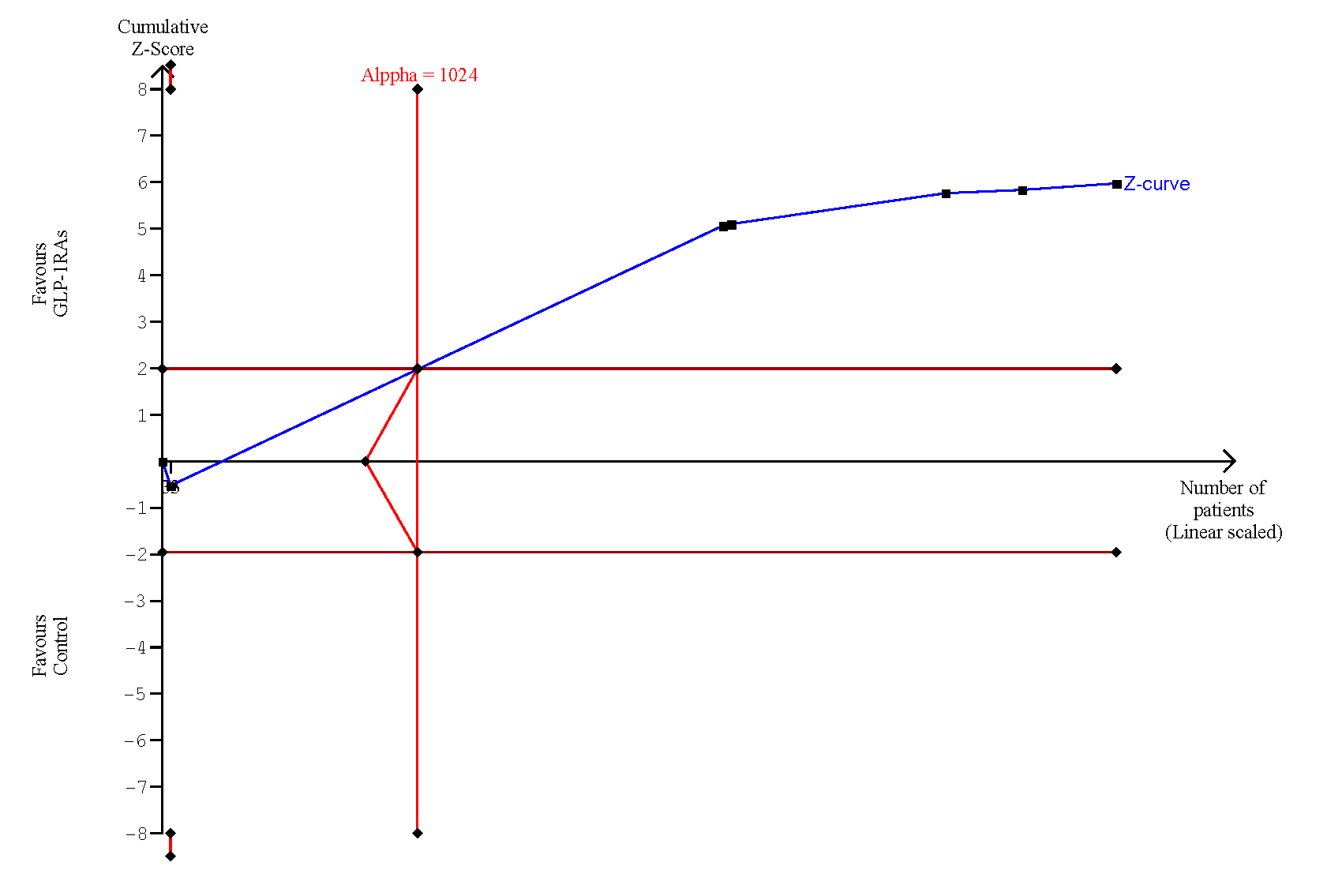
Figure S3.** Trial sequential analysis of the incidence of new-onset diabetes.


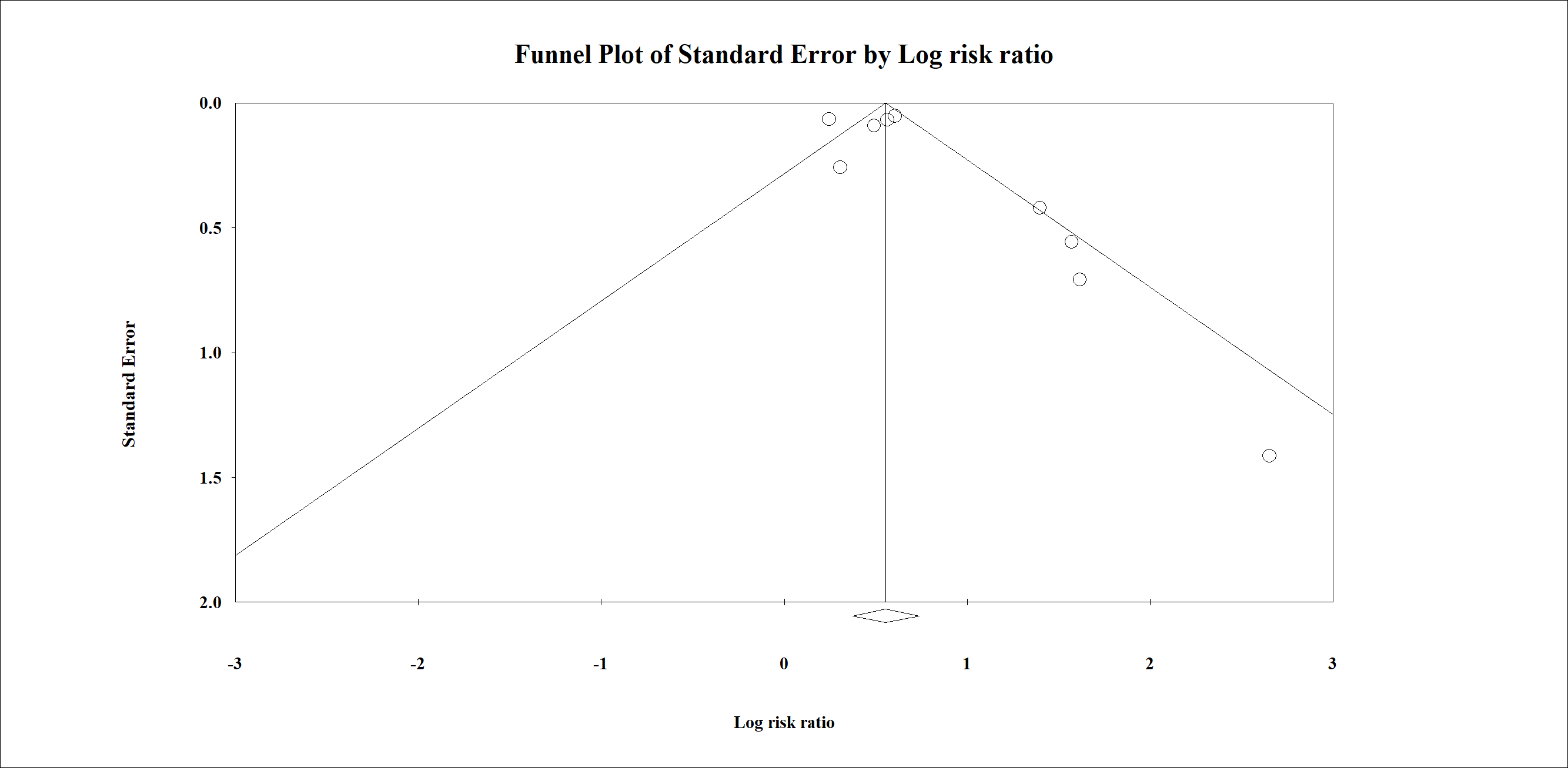


**Figure S4.** Funnel plot of the publication bias of prediabetes reversion to normoglycemia.


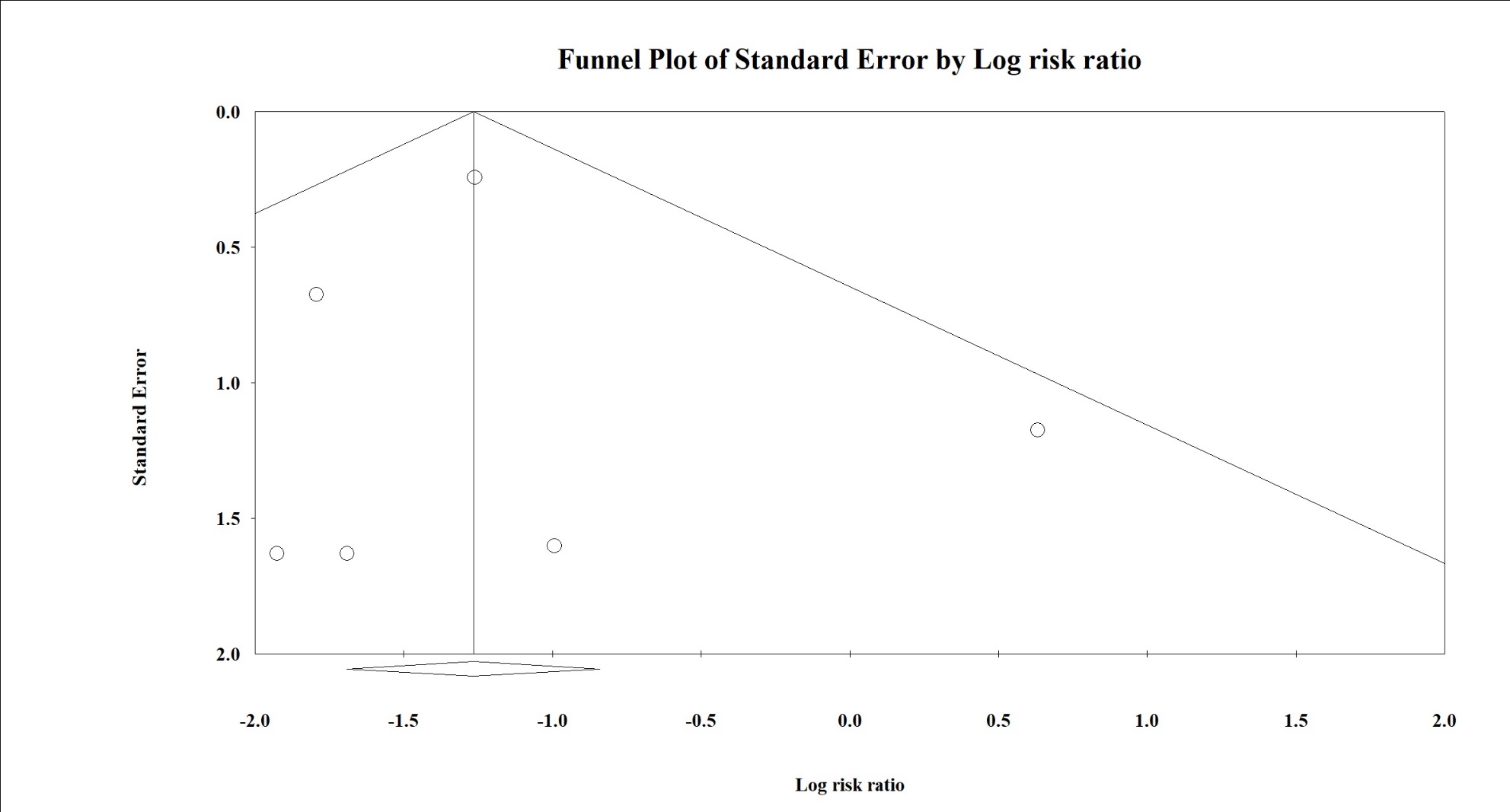


**Figure S5.** Funnel plot of the publication bias of new-onset diabetes.


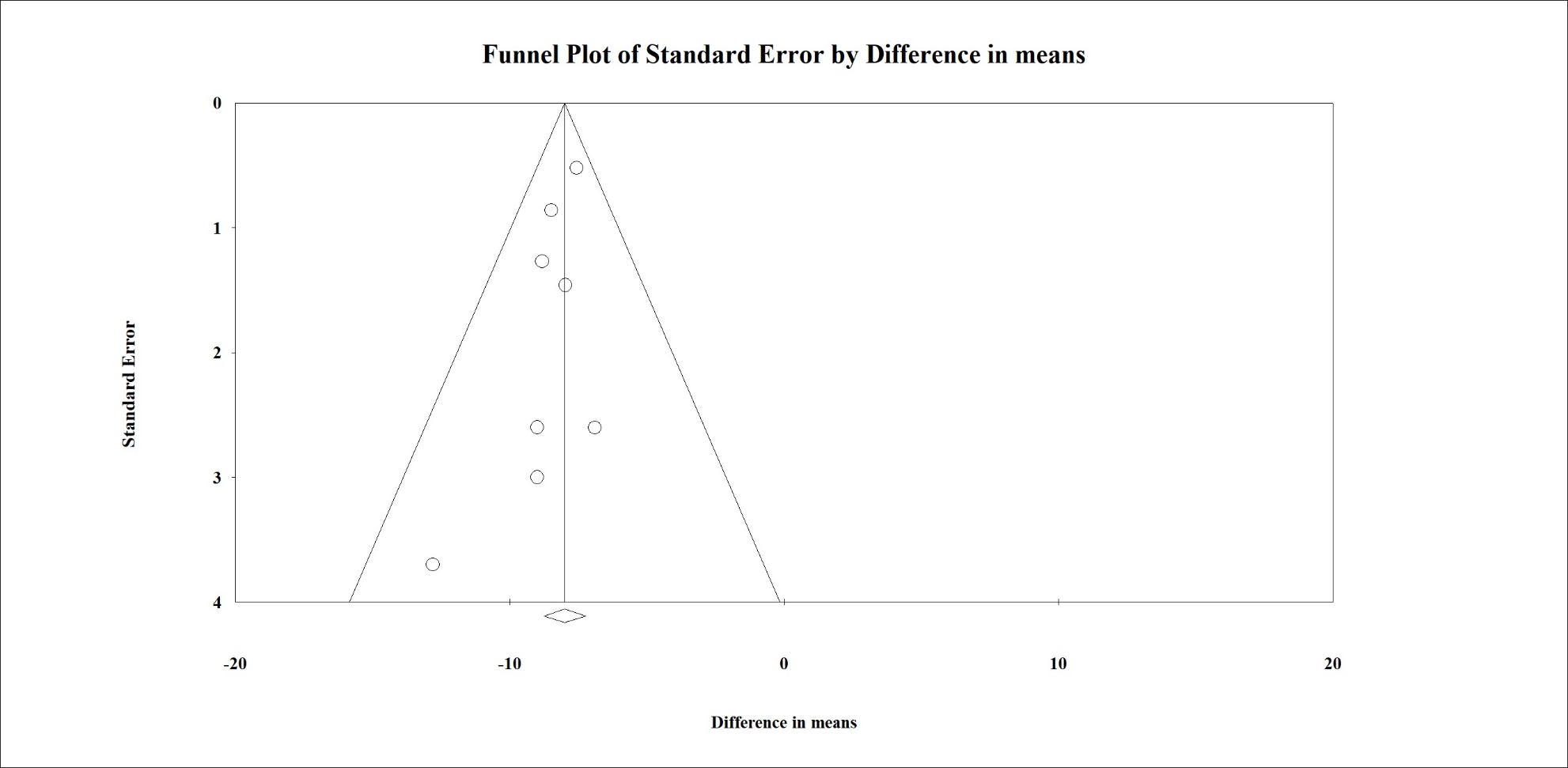


**Figure S6.** Funnel plot of the publication bias of FPG.


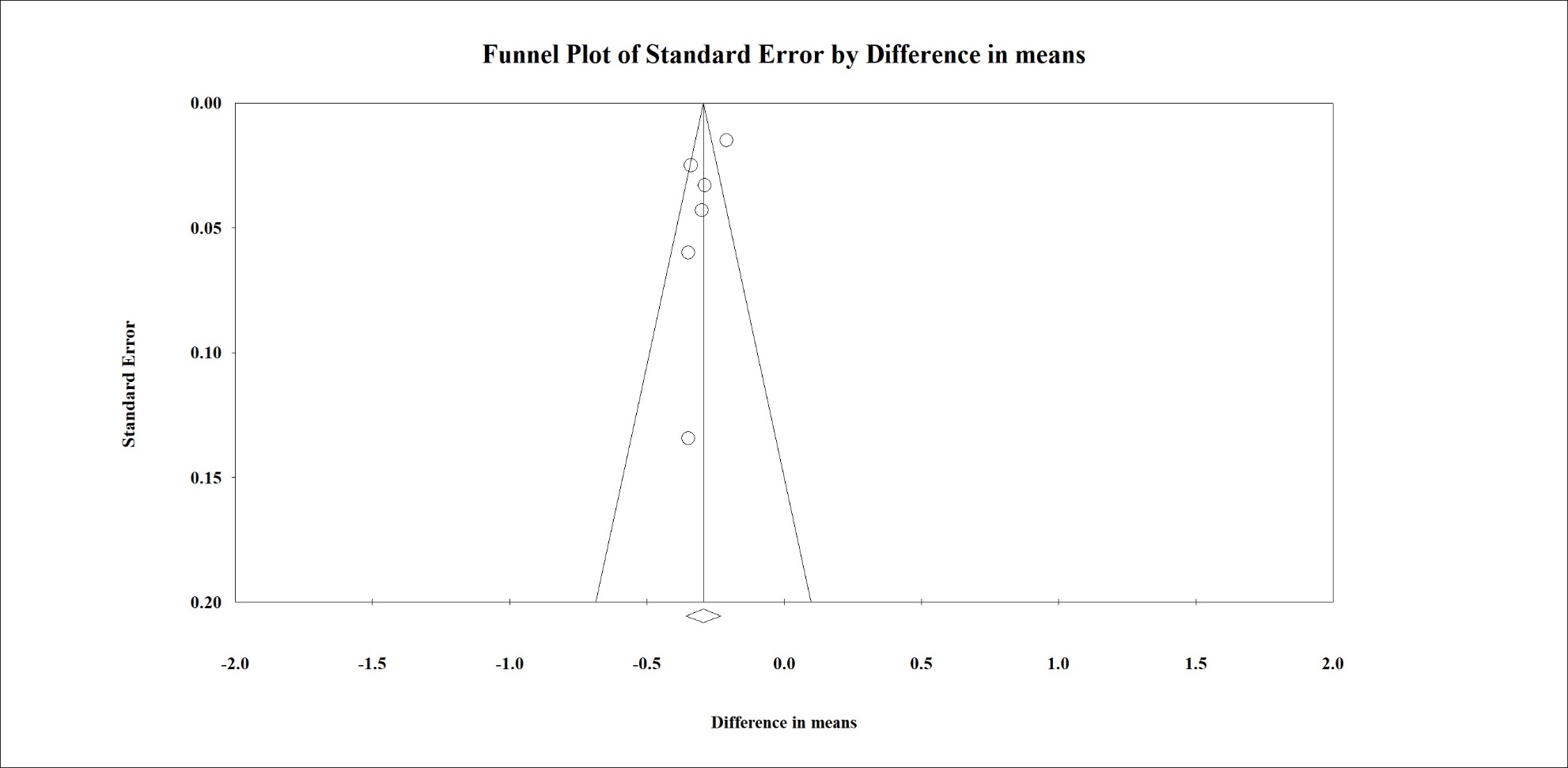


**Figure S7.** Funnel plot of the publication bias of HbA1c.


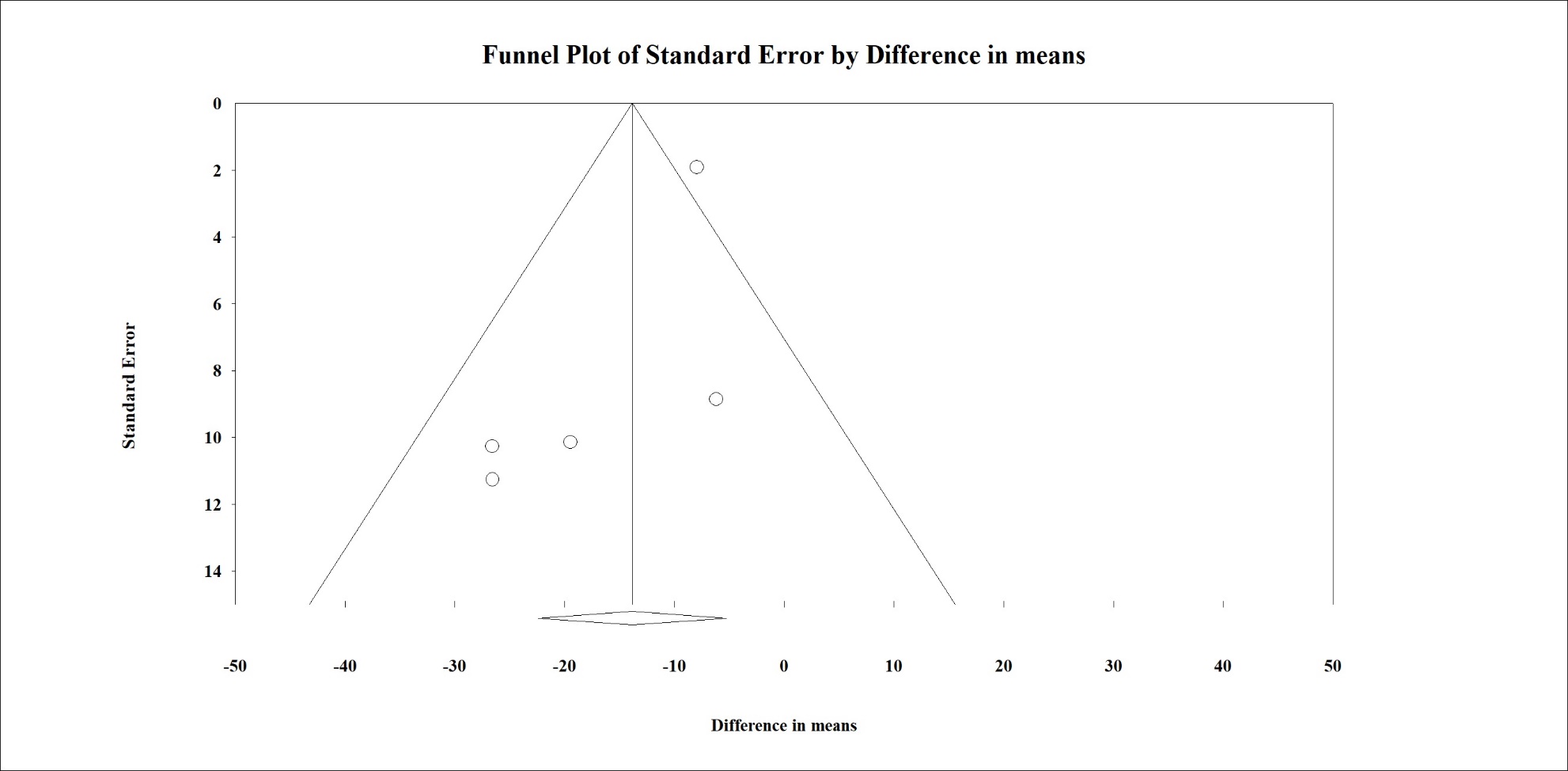
**Figure S8.** Funnel plot of the publication bias of triglycerides.


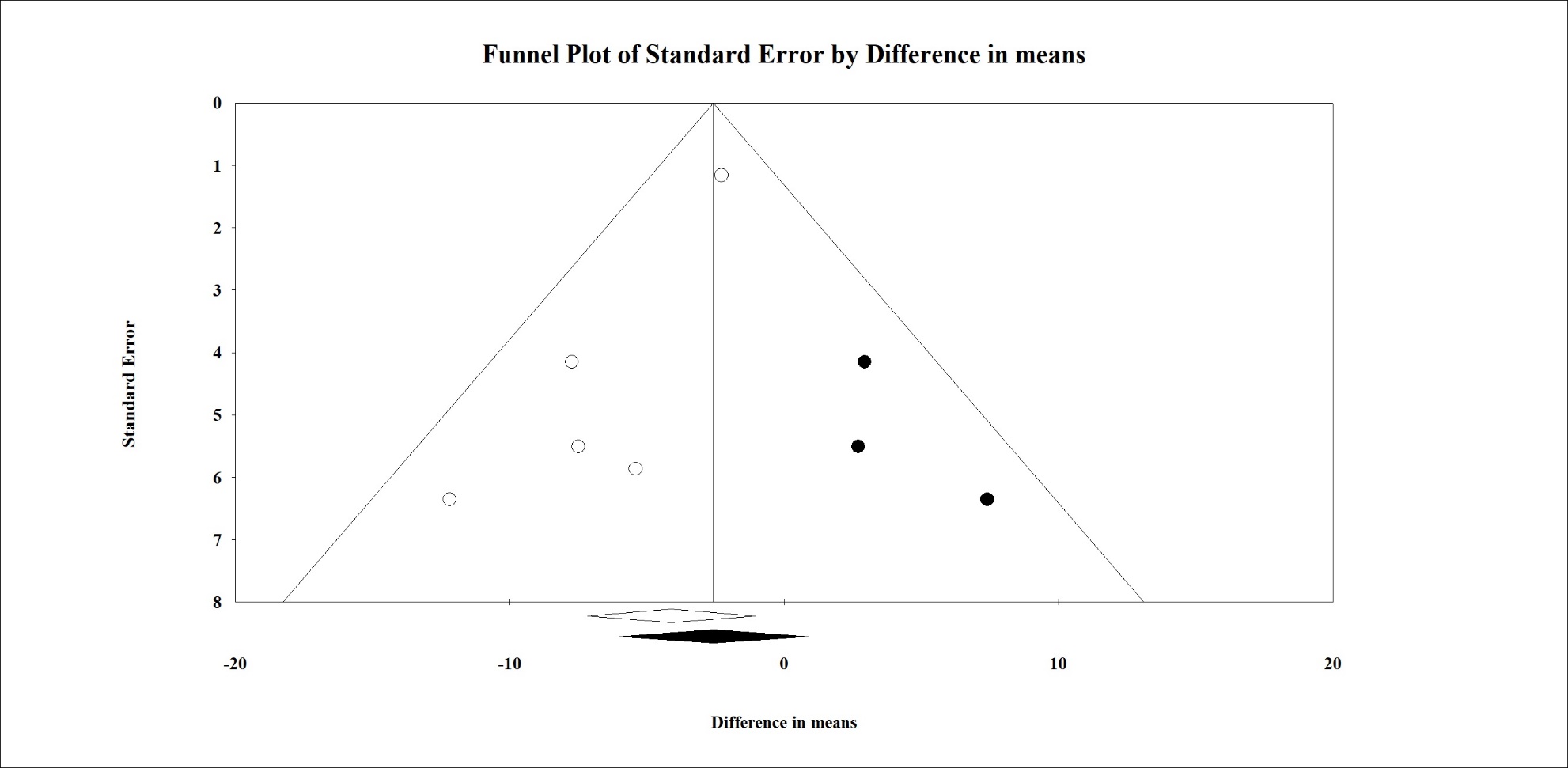
**Figure S9.** Funnel plot of the publication bias of LDL.


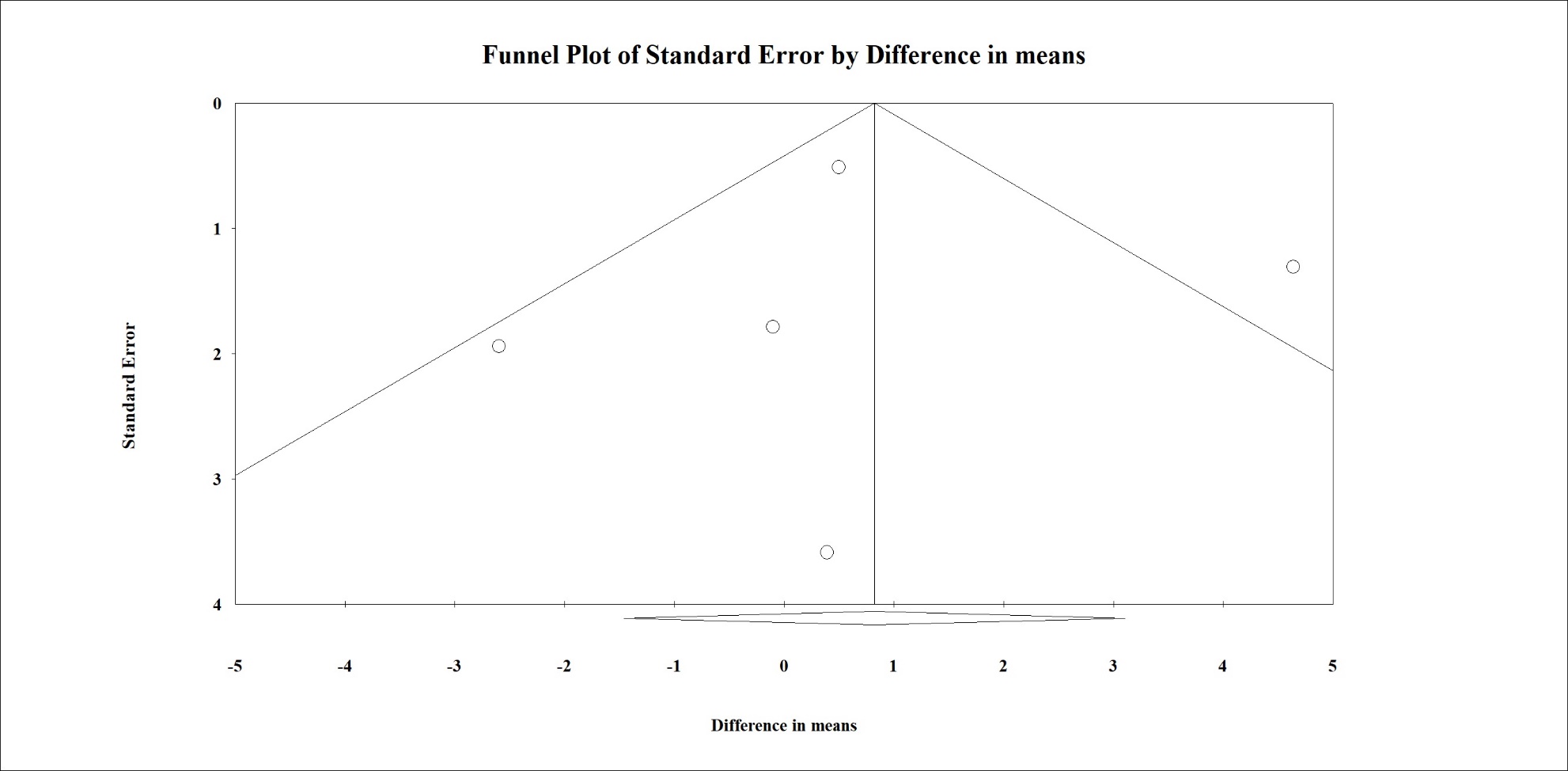


**Figure S10.** Funnel plot of the publication bias of HDL.


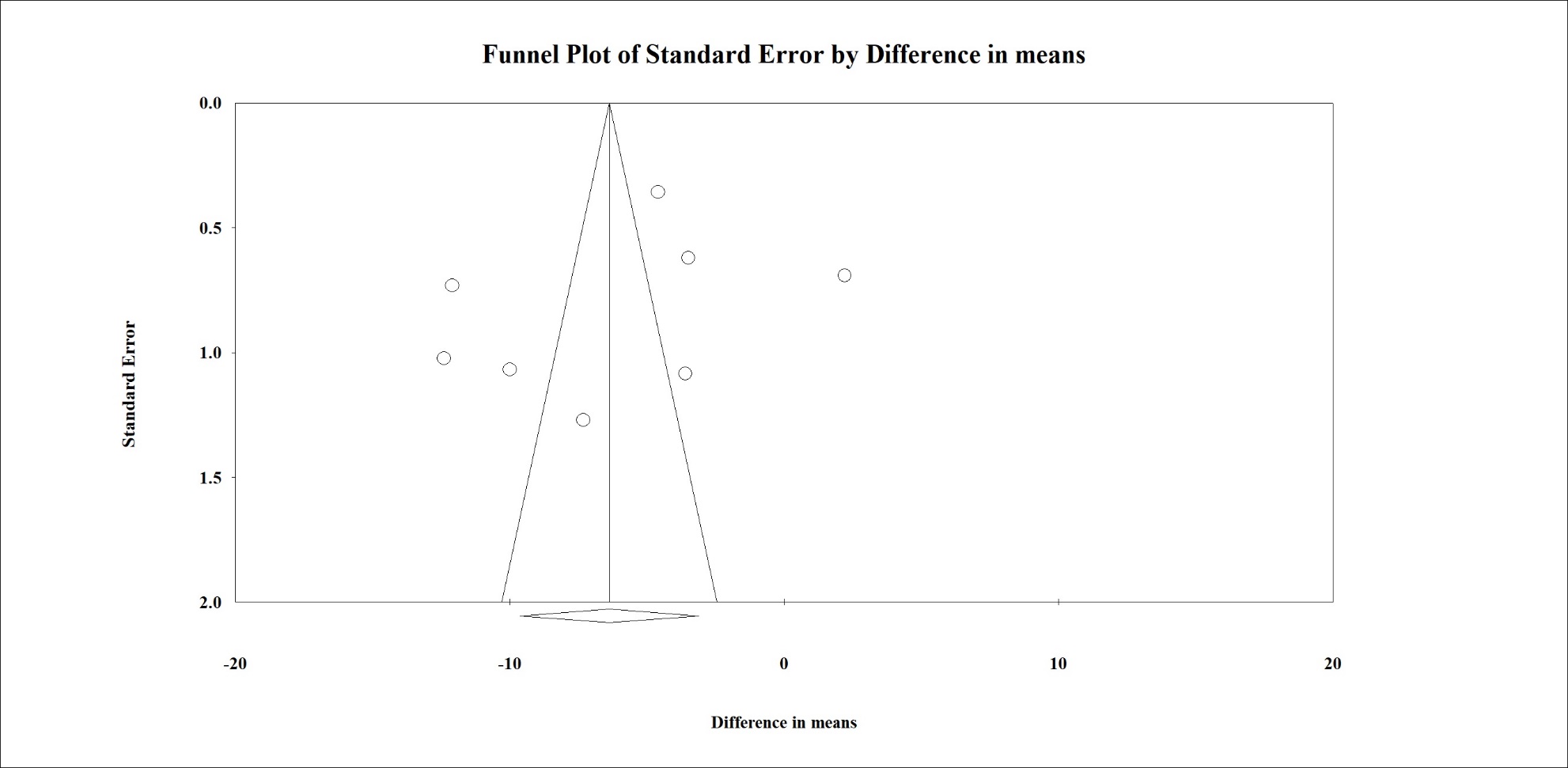


**Figure S11.** Funnel plot of the publication bias of body weight change.


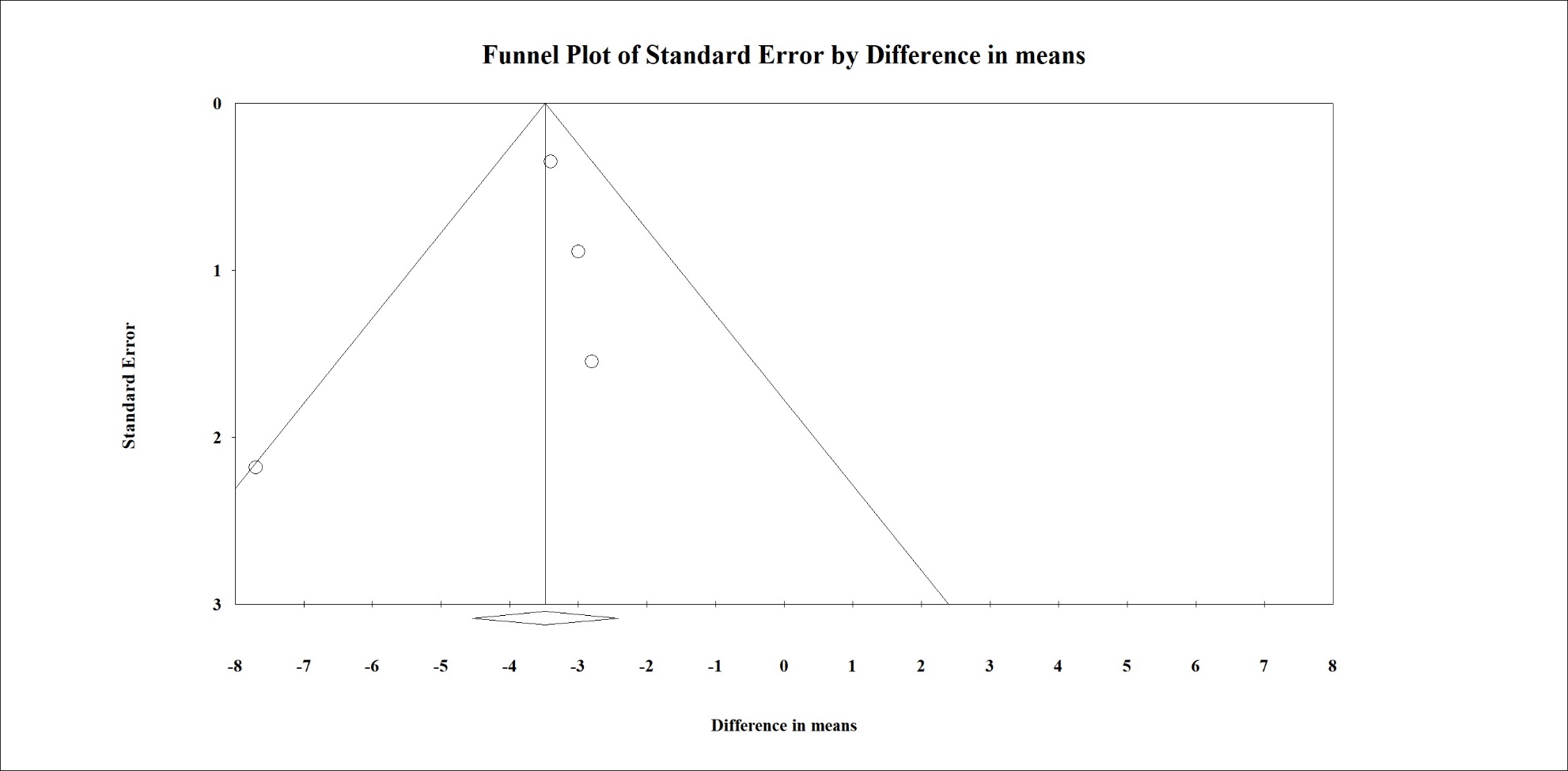
**Figure S12.** Funnel plot of the publication bias of waist circumference.
